# Structural Insights into Viral Determinants of Nematode Mediated *Grapevine fanleaf virus* Transmission

**DOI:** 10.1371/journal.ppat.1002034

**Published:** 2011-05-19

**Authors:** Pascale Schellenberger, Claude Sauter, Bernard Lorber, Patrick Bron, Stefano Trapani, Marc Bergdoll, Aurélie Marmonier, Corinne Schmitt-Keichinger, Olivier Lemaire, Gérard Demangeat, Christophe Ritzenthaler

**Affiliations:** 1 Institut National de la Recherche Agronomique, INRA/UDS UMR 1131, Colmar, France; 2 Institut de Biologie Moléculaire des Plantes, CNRS/UDS UPR2357, Strasbourg, France; 3 Institut de Biologie Moléculaire et Cellulaire, CNRS/UDS UPR 9002, Strasbourg, France; 4 Université de Montpellier 1, Université de Montpellier 2, CNRS UMR 5048, Centre de Biochimie Structurale, Montpellier, France; 5 INSERM, Unité 554, Centre de Biochimie Structurale, Montpellier, France; University of Kentucky, United States of America

## Abstract

Many animal and plant viruses rely on vectors for their transmission from host to host. *Grapevine fanleaf virus* (GFLV), a picorna-like virus from plants, is transmitted specifically by the ectoparasitic nematode *Xiphinema index*. The icosahedral capsid of GFLV, which consists of 60 identical coat protein subunits (CP), carries the determinants of this specificity. Here, we provide novel insight into GFLV transmission by nematodes through a comparative structural and functional analysis of two GFLV variants. We isolated a mutant GFLV strain (GFLV-TD) poorly transmissible by nematodes, and showed that the transmission defect is due to a glycine to aspartate mutation at position 297 (Gly297Asp) in the CP. We next determined the crystal structures of the wild-type GFLV strain F13 at 3.0 Å and of GFLV-TD at 2.7 Å resolution. The Gly297Asp mutation mapped to an exposed loop at the outer surface of the capsid and did not affect the conformation of the assembled capsid, nor of individual CP molecules. The loop is part of a positively charged pocket that includes a previously identified determinant of transmission. We propose that this pocket is a ligand-binding site with essential function in GFLV transmission by *X. index*. Our data suggest that perturbation of the electrostatic landscape of this pocket affects the interaction of the virion with specific receptors of the nematode's feeding apparatus, and thereby severely diminishes its transmission efficiency. These data provide a first structural insight into the interactions between a plant virus and a nematode vector.

## Author Summary

Numerous pathogenic viruses from animals and plants rely on vectors such as insects, worms or other organisms for their transmission from host to host. The reasons why certain vectors transmit some viruses but not others remain poorly understood. In plants, *Grapevine fanleaf virus* (GFLV), a major pathogen of grapes worldwide and its specific vector, the dagger nematode *Xiphinema index*, provides a well-established model illustrating this specificity. Here, we determined the high-resolution structures of two GFLV isolates that differ in their transmissibility. We show that this difference is due to a single mutation in a region exposed at the outer surface of the viral particles. This mutation does not alter the conformation of the particles but modifies the distribution of charges within a positively-charged pocket at the outer surface of virions which likely affects particle retention by *X. index* and, thereby also transmission efficiency. Therefore, we propose that this pocket is involved in the specific recognition of GFLV by its nematode vector. This work paves the way towards the characterization of the specific compound(s) within the nematodes that trigger vector specificity and provides novel perspectives to interfere with virus transmission.

## Introduction

Efficient transmission from host to host by vectors is an important biological feature shared by many animal and plant viruses. Arthropods transmit many viruses to mammals and plants. Examples include highly pathogenic viruses such as *Rift Valley fever virus*, *Dengue virus* or *Chikungunya virus,* primarily transmitted to animals and humans by *Aedes* spp. mosquitoes [1], [2], *Tick-borne encephalitis virus* transmitted by ticks [3] or Sharka/plum pox virus disease affecting stone fruits and vectored by aphids. In animals, transmission by vectors is limited to some genera such as *Alphavirus Flavivirus, Rhabdovirus* or *Reoviridae* and requires a replication cycle in the vector [4]. In contrast, nearly all plant viruses depend on vectors for their transmission. Non-enveloped viruses - the vast the majority of all plant viruses - are generally specifically acquired by their vectors, but do not replicate in them [5], [6], [7], [8].

Over the years, virus transmission has gradually been recognized as a specific process but the molecular mechanisms governing the recognition between a virus and its vector are far from being unraveled. Comparative studies of transmissible and non-transmissible plant virus isolates have led to the identification of determinants in capsid proteins (CP) [9], [10], [11], [12]. In addition to the CP, some viruses require additional viral proteins referred to as helper components for their transmission by vectors (HC) [7], [8], [13]. HCs are viral proteins capable of engaging interactions with the viral CP and putative receptor molecules from the vector. Thus, they act as bridging molecules.

Various motifs in CPs or HCs required for transmission are described for a broad range of plant viruses, in particular members of the genera *Potyvirus*, *Caulimovirus* and *Cucumovirus* vectored by aphids. For example, the rod shaped potyviruses have DAG and PTK motifs in their CP and HC-pro, respectively [14], [15], [16]. In contrast, in the icosahedral *Cucumber mosaic virus* (CMV), the CP is the sole viral determinant of transmission [17]. There, the CP that folds into ß-barrel domains exposes a conserved and negatively charged βH-βI loop exposed at the surface of the virion to establish electrostatic interactions with components inside the aphid's mouthparts [18], [19]. In *Cauliflower mosaic virus* (CaMV), transmission necessitates two HC proteins named P2 and P3 in addition to the CP. Together these proteins form a transmissible viral complex whose assembly depends on interactions between coiled-coil domains [20], [21], [22] and components of the host plants [23]. This complex is thought to be specifically retained in the acrostyle, a specialized anatomical structure in the aphid stylet where virus receptor proteins accumulate [24], [25].

Less is known about the transmission by ectoparasitic nematodes of soil-borne viruses belonging to the genera *Nepovirus* and *Tobravirus*. In the rod-shaped tobraviruses, the partly unstructured C-terminal tail of the CP is necessary but not sufficient to promote transmission and other viral proteins may act as HC [26], [27], [28]. In nepoviruses, the CP that assembles into icosahedral particles is the sole viral determinant involved in transmission specificity, as shown for *Grapevine fanleaf virus* (GFLV) and *Arabis mosaic virus* (ArMV) which are transmitted by two different species of *Xiphinema* nematodes, *X. index* and *X. diversicaudatum*, respectively [29], [30]. Recently, a 3D homology model of GFLV based on the crystal structure of *Tobacco ringspot virus* (TRSV) [31], revealed the existence of a stretch of 11 amino acids within the BC loop of the B-domain that differs between GFLV and ArMV. The transmission of GFLV by *X. index* was abolished when this sequence was replaced by the corresponding region from ArMV. Hence, this loop has all the properties of a determinant for GFLV transmission [32].

The general feature that emerges from all these analyses is that transmission of non-circulative plant viruses involves well-defined and precise interactions between viral and vector molecules. In this respect, parallels can be established with virus-receptor interactions used by animal viruses to enter host cells [33]. However, our current knowledge of the vector-assisted transmission of animal or plant viruses lags far behind that of animal virus-receptor interaction whose details are known in some cases up to the atomic resolution. In the coming years the challenge will be to characterize the key molecules of the vectors engaged in transmission and to gain high-resolution structural insights into their interactions with the cognate viruses.

To understand the molecular details controlling virus-vector interactions, we have use the model pathosystem GFLV - *X. index*. Here, we have identified a GFLV variant (GFLV-TD) poorly transmitted by *X. index* that differs from its parent strain (GFLV-F13) by a single Gly_297_Asp mutation. Using X-Ray crystallography in combination with cryo-electron microscopy 3D reconstruction, we solved the crystal structures of GFLV-TD and GFLV-F13 at 2.7 Å and 3.0 Å resolution, respectively. These 3D structures highlighted the dramatic effect of a single amino acid substitution in GFLV transmission and helped identify a pocket at the virus surface with predicted function in the specific recognition of GFLV by *X. index*. Altogether, the presented results give a first structural insight into the molecular mechanism needed for the specific binding of a plant virus to its nematode vector.

## Results

### Identification and characterization of a GFLV variant defective in nematode transmission

**Figure 1:**
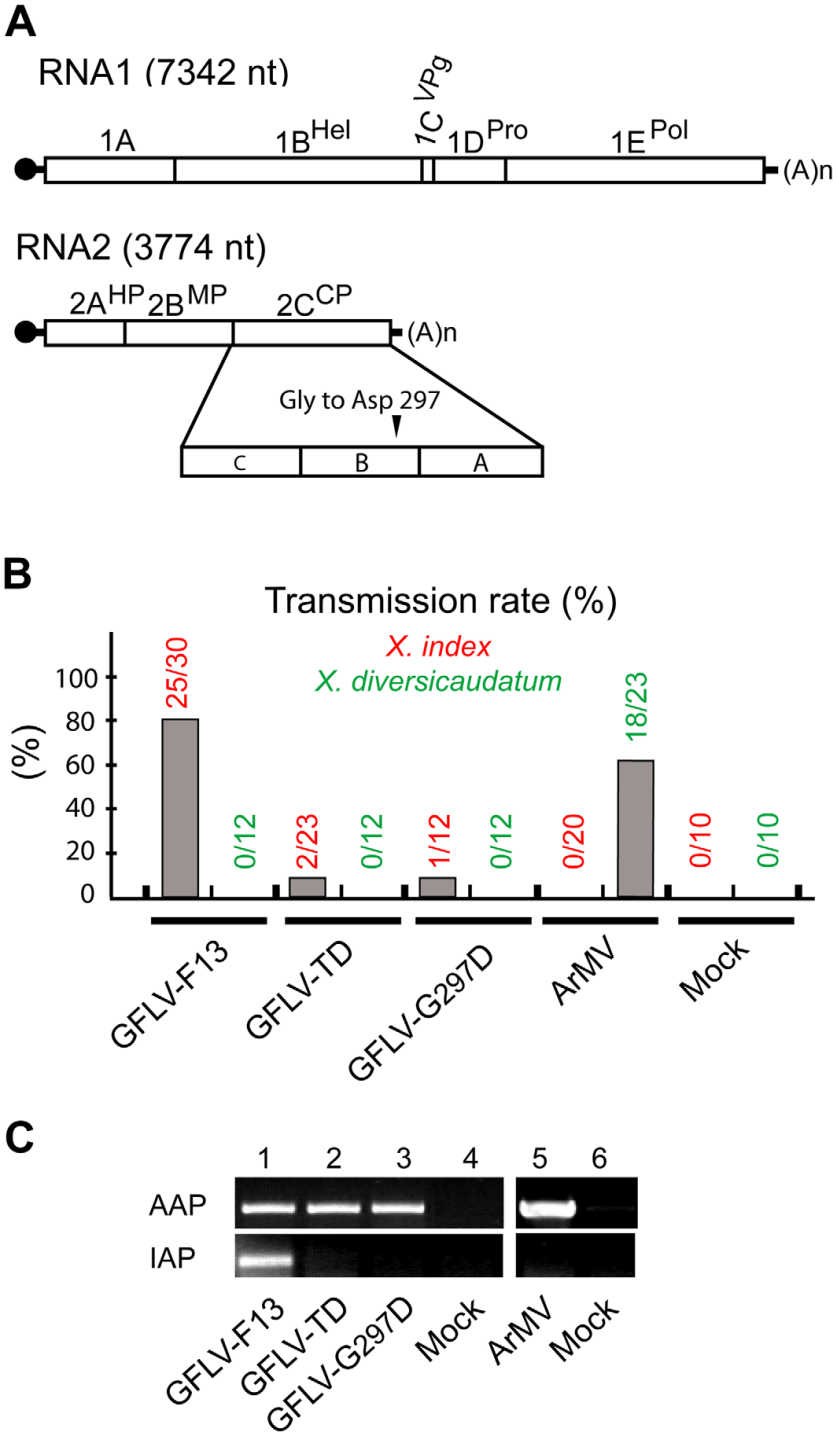
Involvement of capsid protein residue 297 in nematode transmission. **(A)** Genomic organization of GFLV. The 5′ and 3′ untranslated regions are denoted by single lines and the VPg is represented by a black circle. Polyproteins encoded by RNA1 and RNA2 are cleaved in five (1A–1E) and three (2A–2C) final maturation products (open boxes), respectively. 1B, helicase (Hel); 1C, viral protein genome-linked (VPg); 1D, protease (Pro); 1E, RNA-dependent RNA polymerase (Pol); 2A, homing protein (HP); 2B, movement protein (MP) and 2C, coat protein (CP). As indicated, the CP is composed of three domains called C, B, and A. In the variant GFLV-TD, the CP residue Gly at position 297 is replaced by Asp. **(B)** Transmission of wild type GFLV-F13, GFLV-TD and GFLV-G_297_D (the two latter with a Gly^297^ to Asp^297^ substitution) and wild type ArMV by *X. index* and *X. diversicaudatum*. Transmission rates are expressed as the percentage of ELISA-positive plants. **(C)** Virus detection in *X. index* at the end of the AAP and the IAP showed that the mutated viruses and ArMV were ingested but not retained by nematodes. Thirty nematode specimens exposed to source plants infected with GFLV-F13 (lane 1), GFLV-TD (lane 2), GFLV-G_297_D (lane 3), ArMV (lane 5), or mock inoculated plants (lanes 4 and 6) were randomly collected and tested by RT-PCR with GFLV (lanes 1–4) or ArMV (lanes 5 and 6) specific primers. DNA products were analyzed by electrophoresis on 1.5% agarose gels.

GFLV strain F13 (GFLV-F13) was first isolated from an infected grapevine in southern France in 1964 [34]. In agreement with its classification in the *Nepovirus* genus, it contains a bipartite, linear, single stranded positive sense RNA genome. RNA1 plays an essential role in replication and RNA2 is necessary for movement and encapsidation (**Figure 1A**). Ever since its isolation, GFLV-F13 was propagated by mechanical inoculation of the systemic herbaceous host *Chenopodium quinoa*. After four decades of successive passages onto *C. quinoa*, the nematode transmission of varied GFLV-F13 inocula was assessed. This led to the identification of a variant poorly transmitted by *X. index* named GFLV-TD (**Figure 1B**). Beside the defect in transmissibility, GFLV-TD was indistinguishable from its wild-type parental strain GFLV-F13 in terms of symptom development on *C. quinoa*, reactivity to GFLV antibodies in DAS-ELISA and virus purification yields (data no shown). Similarly, in transmission assays (**Figure S1**), no difference in the ability of *X. index* to ingest GFLV-F13 and GFLV-TD was detected by RT-PCR after a monthly acquisition access period (AAP) (**Figure 1C**, **top panel**). However, at the end of the inoculation access period (IAP), GFLV-TD was not detectable by RT-PCR in *X. index* (**Figure 1C**, **bottom panel**), suggesting that it is poorly or not retained by nematodes. These results were consistent with the transmission deficiency of GFLV-TD likely due to the paucity or incapacity of the virus to be retained by the vector at specific sites within its feeding apparatus.

Since the CP is the sole determinant required for GFLV transmission [29], [30], the GFLV-TD CP coding sequence was characterized by IC-RT-PCR and sequencing to identify potential amino acid mutations. A single Gly to Asp mutation at position 297 was found. To assess whether this mutation explained the deficiency in nematode transmission of GFLV-TD, it was introduced into the GFLV-F13 RNA2-encoded CP gene by site-directed mutagenesis of the corresponding cDNA infectious clone [35]. Similar to the natural GFLV-TD variant, the site-directed mutant, named GFLV-G_297_D, was poorly transmitted by *X. index* (**Figure 1B**). In addition, GFLV-G_297_D was not retained by the vector after the IAP, therefore mimicking GFLV-TD (**Figure 1C**). These results confirm the critical role of Gly^297^ in GFLV transmission efficiency.

### GFLV-F13 and GFLV-TD structures

To determine their atomic structures, GFLV-TD and GFLV-F13 virions were crystallized as described [36]. Two crystal forms were obtained and analyzed (**Table 1**). The asymmetric unit of the GFLV-TD crystal (PDBid 2Y26) contains 20 CP subunits and that of GFLV-F13 contains 60 subunits, *i.e.* the entire virion. The structure of GFLV-TD was solved by molecular replacement using a cryo-electron microscopy model at 16.5 Å resolution (**Figure S2**) followed by solvent flattening, non crystallographic symmetry (NCS) averaging and refinement at 2.7 Å (**Table 1**). The complete GFLV-TD particle was generated by symmetry operations and used as a model to solve the structure of GFLV-F13 (PDBid 2Y7T, 2Y7U, 2Y7V) by molecular replacement at 3.0 Å (**Table 1**).

**Table 1:** Crystallographic analysis of GFLV particles.

Virus	GFLV-TD	GFLV-F13
**Data collection statistics** £		
Beamline	ESRF/BM30	SLS/X06DA
Space group (number)	P2_1_3 (198)	P1 (1)
Unit cell lengths a, b, c (Å)	408.0	279.4 279.5 293.3
Unit cell angles alpha, beta, gamma (°)	90.0	102.4 116.4 108.2
Resolution range (Å)	36 – 2.7	135 – 3.0
Highest resolution shell (Å)	2.77 – 2.7	3.08 – 3.0
No. of unique reflections	563009 (32448)	1214336 (73170)
Completeness (%)	92.0 (72.0)	88.1 (71.7)
Multiplicity	11.0 (3.1)	2.0 (1.9)
R_merge_ (%)†	12.5 (68.1)	10.0 (35.0)
*<I/sigma(I)>*	18 (1.9)	9.2 (2.4)
**Molecular replacement**		
Resolution range (Å)	30 – 15	15 – 6.0
Asymmetric unit content	20-mer	60-mer
Model	EM map	GFLV-TD
Correlation/R-factor(%)#	60.4/56.4	70.6/34.7
**Refined atomic structure**		
Resolution range (Å)	36 – 2.7	135 – 3.0
R-factor/R-free (%)*	19.3/21.0	19.0/20.7
Number of capsid and solvent atoms	79100/556	237060/–
Protein and solvent ADPs (Å^2^)**	40.9/36.9	35.7/–
R.m.s.d. on bonds (Å) and angles (°)	0.009/1.19	0.010/1.20

**£:** Statistics are given for reflections with I> = 0 and values in parentheses are for the highest resolution shell.

†R_merge_ = σ*_hkl_* σ*_i_* |*I_i_(hkl)* - <*I(hkl)*>|/σ*_hkl_* σ*_i_ I_i_(hkl)*.

# The high R-factor can be explained, among other reasons, by the model used (a low resolution EM reconstruction, without filtering) and the absence of a bulk solvent correction.

*The cross-validation (R-free) was calculated with 5% of the data.

**ADPs: Atomic displacement parameters.

**Figure 2:**
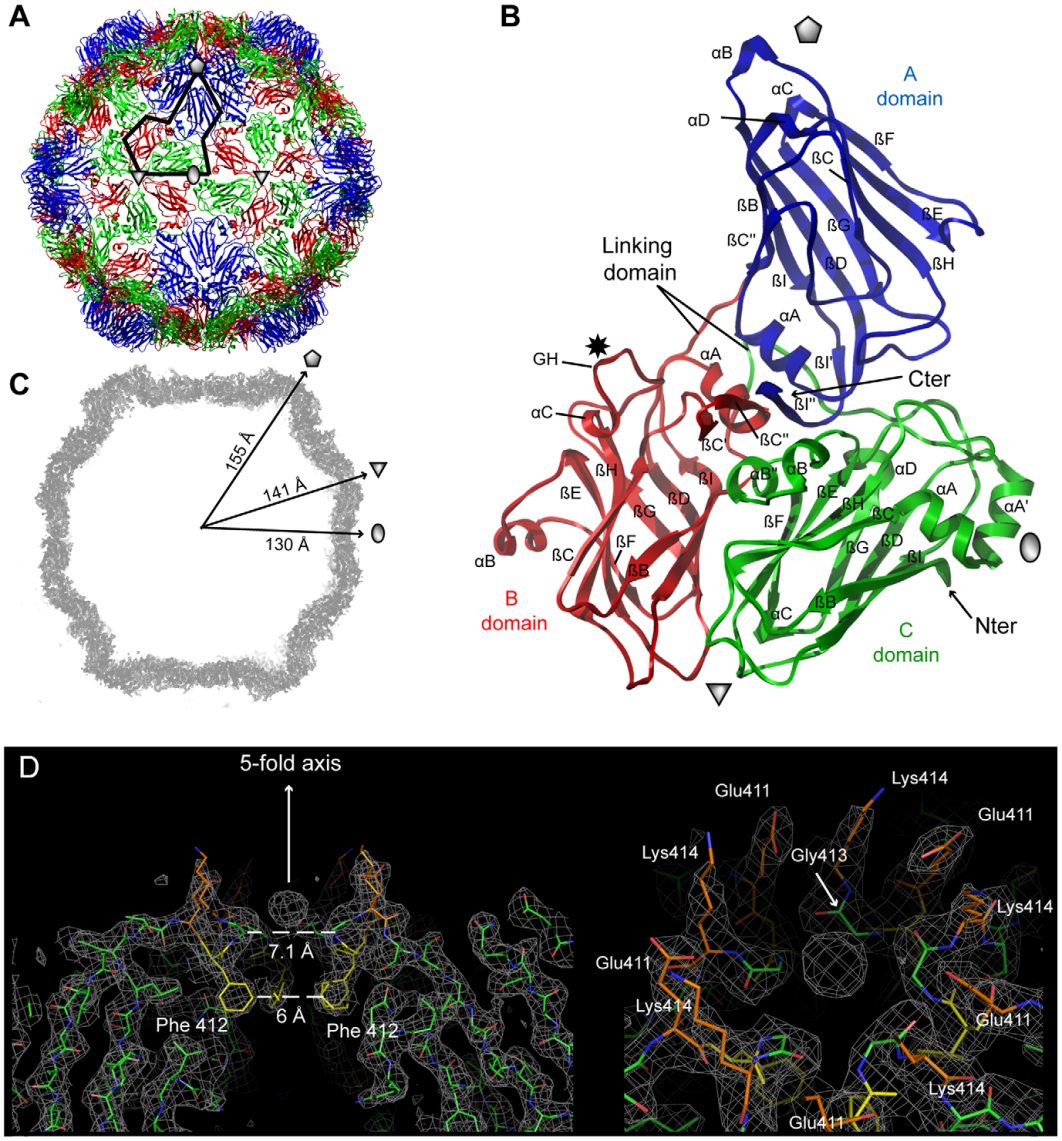
Crystal structures of GFLV-F13 and GFLV-TD. (**A**) The structures of GFLV-F13 and of GFLV-TD are very similar as illustrated by the extremely low r.m.s.d. values (see **Table S1**). For this reason only the highest resolution model (GFLV-TD) is represented in this figure. The ribbon diagram of the virus capsid is viewed down an icosahedral 2-fold axis normal to the plane of the paper. Sixty copies of the CP are arranged in an icosahedral pseudo *T* = 3 symmetry. The black line delineates one CP position. The grey pentagon, triangle and oval symbolize the icosahedral 5-fold, 3-fold and 2-fold symmetry axes, respectively. (**B**) Each CP comprises three jellyroll β sandwiches termed C, B and A domains from the N- to the C-terminus and are depicted in green, red and blue, respectively. A star indicates the position of residue 297. (**C**) The central section of the *2Fo-Fc* electron density map (2 σ contour level) of a GFLV-F13 particle is viewed down a viral 2-fold axis. The outer radial dimensions along the icosahedral symmetry axes are indicated. (**D**) A thin slice of 2*Fo-Fc* electron density map (contoured at 1σ) reveals a strong density peak (about 3 σ in the 2*Fo-Fc* and 17 σ in the *Fo-Fc* map) on the 5-fold axis of GFLV-TD. The arrow symbolizing the axis points towards the outer surface of the particle as well as neighbouring charged residues (Lys^414^ and Glu^411^) whereas Phe^412^ side chains are directed towards the viral cavity. The right panel shows a slightly shifted top view illustrating the organisation of the residues around the 5-fold axis. Five water molecules bridge Gly^413^ carbonyl groups and Asp^411^ side chains to the large central ion, possibly a phosphate coming from the crystallization medium.

**Figure 3:**
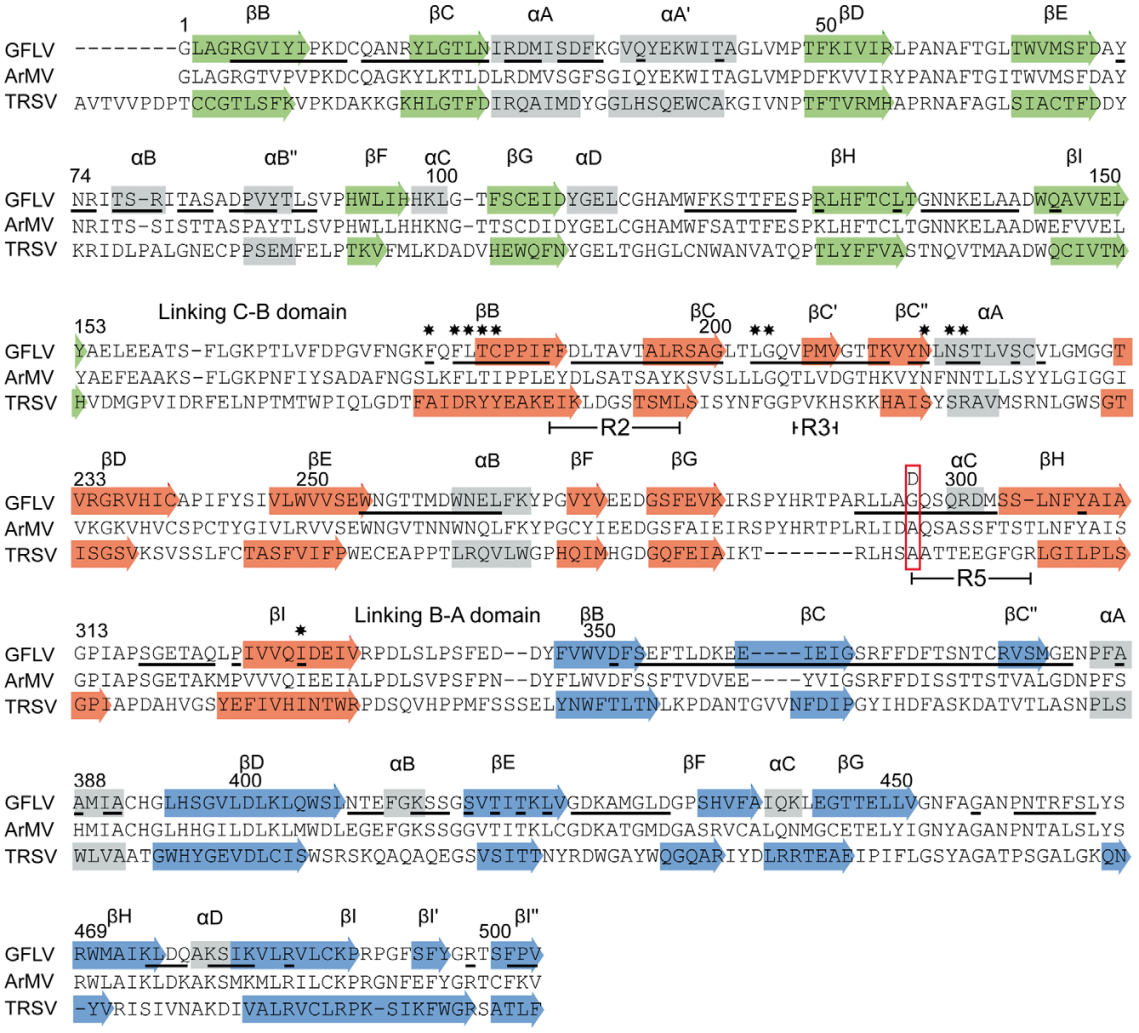
Alignment of the CP amino acid sequences of GFLV, ArMV and TRSV. Secondary structures observed in GFLV and TRSV crystal structures are indicated by arrows (β strands) and grey blocks (α helices). The sequence alignment was created with Clustal X. The same color code as in **Figure 2** is used for strands to indicate the three CP domains: green, red and blue for the C, B, and A domains, respectively. Residues located at the outer surface of the GFLV capsid are underlined. Residues present at position 297 in GFLV-TD, GFLV-F13, ArMV and TRSV are boxed in red. Regions R2, R3 and R5 (see [32] are indicated below the alignments. Stars indicate residues at the bottom of the putative ligand-binding pocket.

In both cases, the icosahedral GFLV capsid is formed by 60 copies of the CP arranged according to a pseudo *T* = 3 symmetry (**Figure 2A**). The CP folds into three jelly-roll β sandwiches. To follow the TRSV nomenclature, the three jelly-roll domains were named C, B, and A from the N- to C- termini, respectively. Two linking peptides connect the C-B and B-A domains (**Figure 2B**). The B and C domains clustered at the 3-fold axis. Five A-domains organized around the 5-fold axis form a protrusion at the capsid's surface (**Figure 2A**). The particle outer radius seen down the 5-fold, 3-fold and 2-fold axes is 155 Å, 141 Å and 130 Å, respectively (**Figure 2C**). The A-domain deviates most from the β sandwich fold of the other domains with an extensive insertion between the βC and the βD strands that comprises one additional strand (**Figure 3**). This is in contrast with the capsid structures of closely related comoviruses where two strands are added at this position [37]. Along each 5-fold axis, *i.e.* the summit of the pentamers, a channel with an inner diameter of 7.1 Å contains an additional electron density that may be attributed to an ion (**Figure 2D**). However, the distance to the neighboring Lys atoms is incompatible with direct hydrogen or ionic bonding (**Figure 2D**), and suggests, in agreement with the presence of surrounding density peaks, that the ion is linked via intermediate water molecules.

The structural variability of CP subunits within a capsid was very low. The average root-mean-square distances (r.m.s.d.) of pair-wise CP superposition were 0.07±0.01 Å and 0.09±0.02 Å for GFLV-TD (20 CPs) and for GFLV-F13 (60 CPs), respectively (**Table S1**). The superposition of the GFLV-F13 asymmetric unit (20 CPs) onto one third of the GFLV-TD caspid as rigid blocks, led to an r.m.s.d. of 0.4 Å for 10080 Cα positions. Higher deviations were found locally with a maximum distance of 1.9 Å at crystal packing contacts. At the level of individual CPs, the two viruses were very similar with an average r.m.s.d. of 0.13±0.01 Å over 504 Cα atoms (**Table S1, Figure S3A**). Overall we could not find any significant conformational change, neither between the two variants, nor inside their respective capsid.

### Comparison of GFLV and TRSV structures

GFLV and TRSV are both transmitted by *Xiphinema* nematodes [38], [39]. As mentioned above, a 3D model of GFLV based on the crystal structure of TRSV helped identify a region at the virion's surface with function in nematode transmission [32]. As expected from CP sequence homology, the CP of GFLV and TRSV display similar 3D architectures with a good superimposition of the CP folds (**Figure S3B**). Both virions have about the same outer dimensions but those of TRSV are slightly smaller than those of GFLV. The greatest capsid radius of TRSV measured down the 5-fold, 3-fold and 2-fold symmetry axes is 155, 137 and 123 Å [31]. Overall contacts between the CP subunits of GFLV are the same as those described for TRSV [31]. Subunit interfaces on the 2-fold and 3-fold axes involve the αA' helix in the C domain and the βHI and βBC loops in the B and C domains, respectively (**Figure S4**). The three jelly-roll domains of the GFLV and TRSV CPs are nearly identical, except for the presence of extra α helices and two supplementary β sheets in the GFLV structure (**Figure 3**). The independent superimposition of the C, B and A domains showed the A is the most divergent and C domains the most conserved (**Table S1**). The most striking difference between TRSV and GFLV is the GH loop located at the outer surface of the B domain. In GFLV this loop is longer and much more prominent than in TRSV (**Figure S3B**). Also, the N-terminal tail facing the interior of the capsid in TRSV is absent in GFLV (**Figure S3B**). This tail accounts almost exclusively for the size differences between the two CPs (504 residues in GFLV vs 513 in TRSV).

### Functional role of residue 297 in transmission

**Figure 4:**
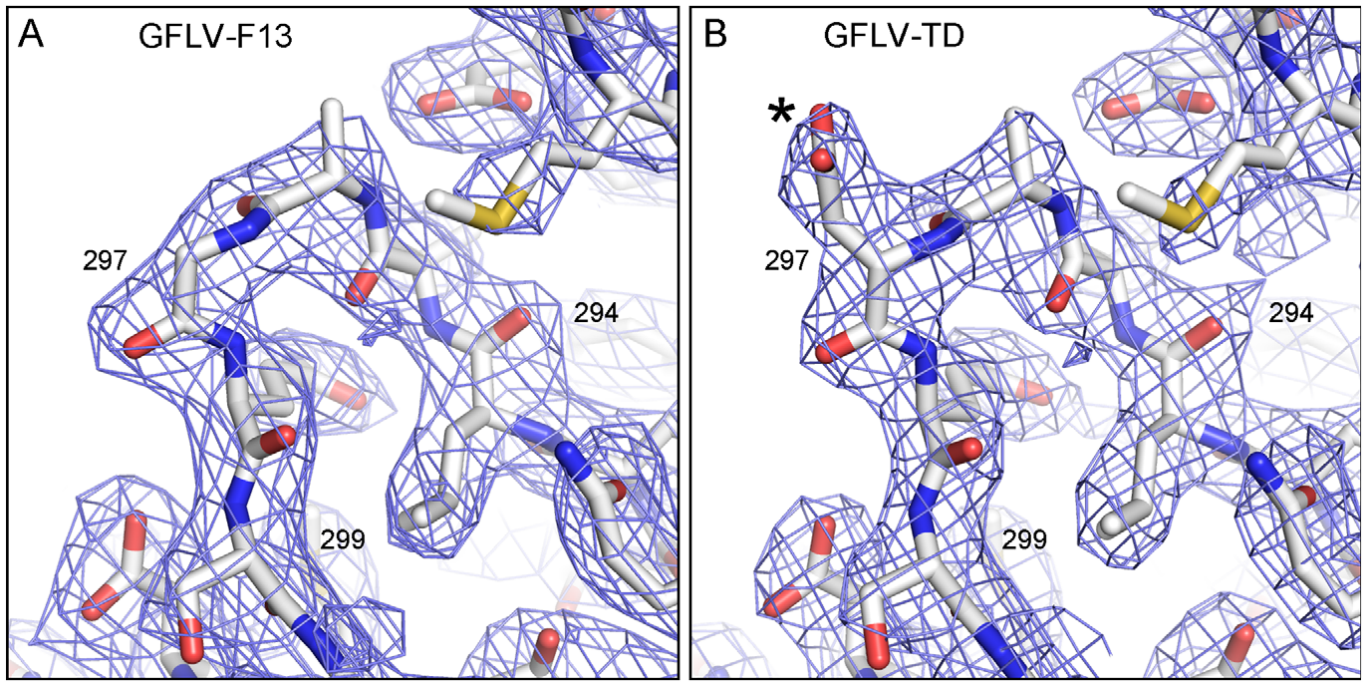
Close-up view of the protruding GH loop within the CP B domain of GFLV-F13 and GFLV-TD. The conformation of the GH loop in GFLV-F13 (**A**) and GFLV-TD (**B**) are identical. The only difference is restricted to the presence of additional electron density corresponding to the Asp^297^ side chain in GFLV-TD in (**B**, star). *2Fo-Fc* electron density maps are contoured at 1.2 σ.

We previously hypothesized that residues important for transmission are exposed at the virion outer surface [32]. According to the GFLV crystal structures, 381 out of 504 CP residues are accessible to the solvent and 208 of them are located at the surface of the virion (underlined in **Figure 3**). Remarkably, among those, residue 297 lies in the most exposed part of the GH loop in the B-domain and is highly accessible to the solvent (**Figure 2B**, **Figure S5**). Sequence information and experimental electron density unambiguously helped identify an Asp side chain at this position in GFLV-TD (**Figure 4**). The conformation of the GH loops in the structures from GFLV-F13 and GFLV-TD is nearly identical with a maximum distance of 0.18 Å between equivalent atoms (**Figure 4**) and therefore, cannot account for the loss of GFLV-TD transmission. Consequently, in the absence of major differences between both structures, the addition of a negatively charged side chain per CP resulting from Gly_297_Asp substitution is presumably responsible for the loss of transmissibility by the nematode.

**Figure 5:**
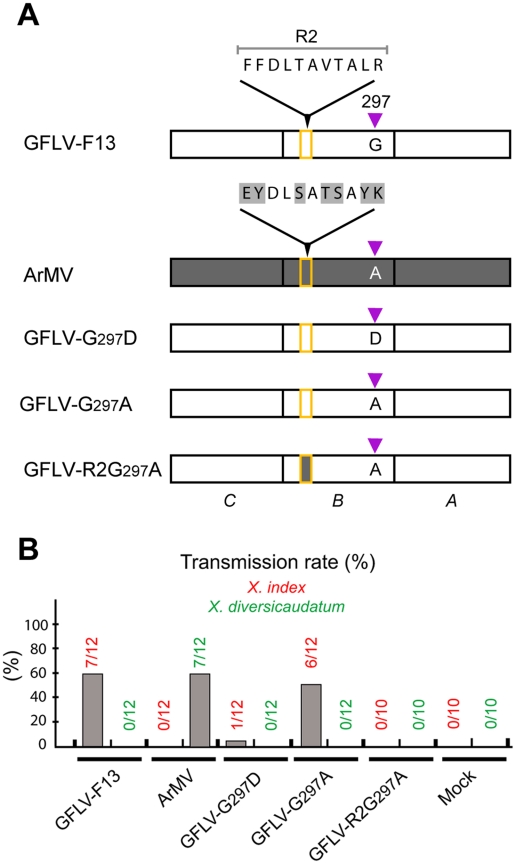
Nematode transmission of GFLV CP mutants. (**A**) The mutants differed in their CP B domain, some of them containing modifications in the R2 region (residues 188 to 198) and/or at position 297. The nature of residue 297 is indicated and the R2 region consisted of GFLV (open rectangle with orange border) or ArMV (grey rectangle with orange border) sequences. The sequence differences are highlighted in grey in the enlargement of ArMV CP R2 region. (**B**) Transmission rate is expressed as the percentage of infected plants over the plants tested.

As mentioned above, a stretch of 11 residues within the CP named region 2 (R2) is essential for GFLV transmission by *X. index* [32]. Knowing that CP amino acid 297 also affects transmission efficiency and that Gly^297^ and R2 are relatively close together (**Figure S5**), we investigated whether both could act synergistically. To address this issue, GFLV amino acid residues in both locations were exchanged by their ArMV counterparts. The single substitution Gly_297_Ala generated a recombinant named GFLV-G_297_A and the dual substitution of R2 and Gly^297^ generated a recombinant named GFLV-R2G_297_A. Transmission assays showed that GFLV-G_297_A was transmitted by *X. index* but not by *X. diversicaudatum* (**Figure 5**). In contrast, GFLV-R2G_297_A was no longer transmitted by either nematode species (**Figure 5**), although virions were ingested by nematodes during AAP (**Figure S6**). These results indicate that Gly^297^ can be substituted by Ala but not by Asp without effect on transmission by *X. index*. Moreover, the simultaneous substitution of Gly^297^ and R2 by ArMV sequences is not sufficient to confer transmission by *X. diversicaudatum*, suggesting that additional residues may be involved.

### Identification of a putative ligand-binding pocket

**Figure 6:**
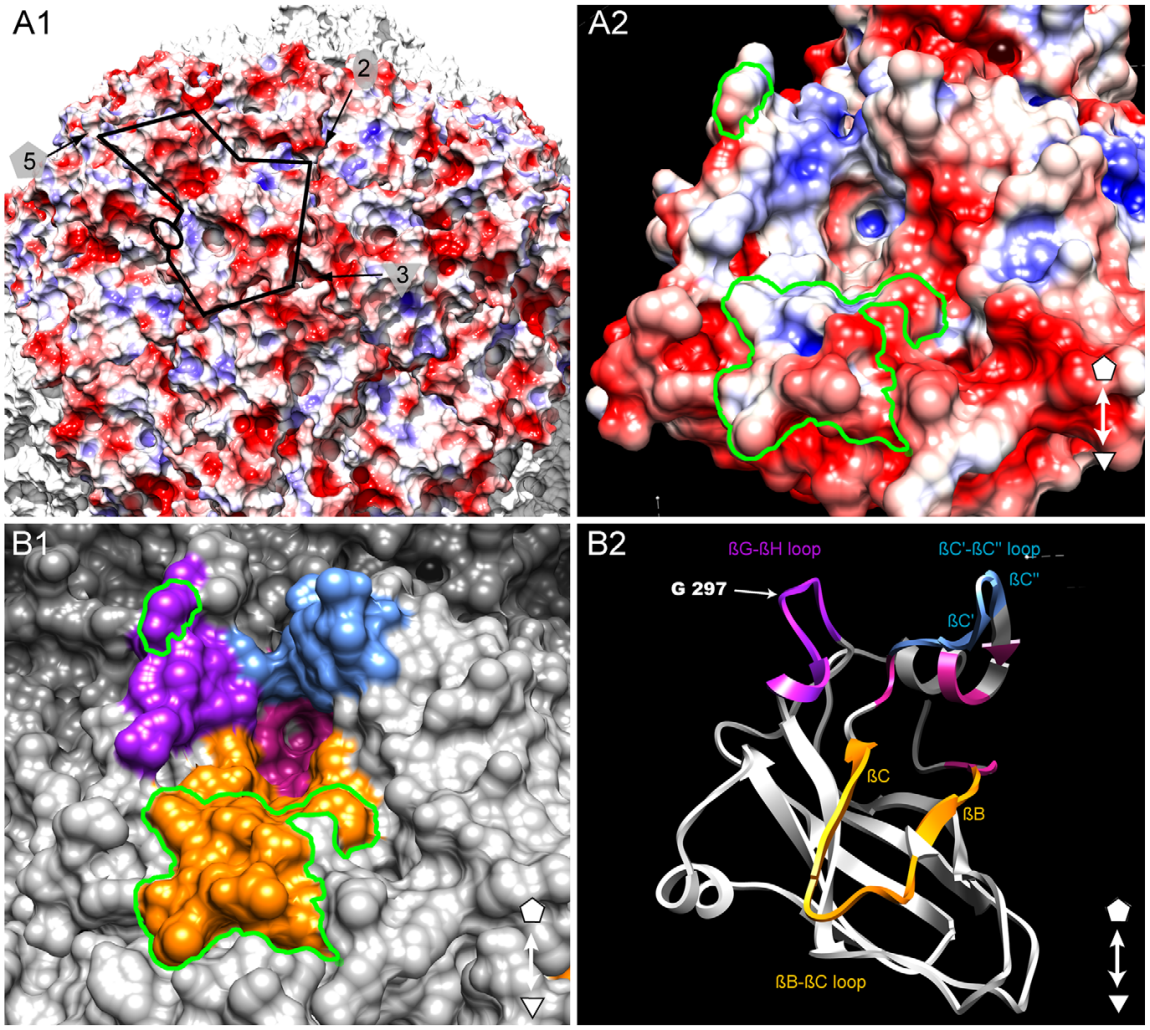
Putative ligand-binding pocket. (**A**) Representation of the GFLV-F13 capsid with red, blue and white regions showing negative, positive and neutral electrostatic surface potentials, respectively. The black line highlights a single CP subunit and arrows denote the 5-, 3-, and 2-fold axes. (**A1**) Residue 297 is indicated by an open black circle on the left side of the positively charged pocket. (**A2**) Detailed view of the positively charged pocket. The electrostatic potential was calculated with APBS and visualized from -3 to 3 k/e^-^ with *Chimera* using a probe of 3 Å to display a smoother surface. (**B**) Top views of the putative ligand-binding pocket at the surface of the GFLV-F13 capsid. (**B1**) View of GFLV-F13 outer-surface residues at the same magnification and orientation than in A2. Residues of the putative ligand-binding pocket are colored using the following color code: GH loop (purple), BC loop (yellow), C′C″ loop (blue) and base of the pocket (red). A green line delineates the Gly^297^ and region R2. (**B2**) Ribbon view of the putative ligand-binding pocket using the same color code as in B1.

The GFLV structure was inspected in the proximity of the residue Gly^297^ and of the region R2 to identify additional residues that may act as transmission determinants. Gly^297^ and R2 are located at the edge of a positively charged pocket within the B-domain, whereas most of the GFLV outer surface is negatively charged (**Figure 6A**). The walls of this pocket are formed essentially by the GH, BC and C′C″ loops encompassing Gly^297^, R2 and the previously defined region R3 [32], respectively (**Figure 6B**). The base of the pocket (**Figure 6B**, purple residues) is formed by at least 11 residues deeply embedded in the capsid shell but still accessible to the solvent (**Figure 3** **, stars**). In the crystal structures of GFLV-F13 and GFLV-TD, the residues of the GH, BC and C′C″ loops are well exposed at the outer surface of the capsid (**Figure 3** **, Figure S5**). This includes the residues Phe^188+189^, Thr^192+195^ and Leu^197^ from R2 which are different between GFLV and ArMV and may participate in the specific binding of GFLV to *X. index* (**Figure 3** and [32]). Altogether, our data suggest that a positively charged pocket located within the GFLV CP B-domain between the 3-fold and 5-fold axes may constitute a ligand recognition site.

## Discussion

GFLV-TD is a natural variant of GFLV-F13 that emerged spontaneously in the greenhouse after multiple mechanical passages in *C. quinoa* plants over time. Loss of virus transmission is not uncommon under such experimental conditions [12], [40], [41], [42], [43]. However, to our knowledge this is the first isolation and characterization of a spontaneously occurring transmission-deficient nepovirus. In the case of GFLV-TD, CP sequencing revealed that a single Gly_297_Asp mutation had occurred. A reverse genetics approach confirmed the involvement of CP residue 297 in the transmission deficiency of GFLV-TD by *X. index*. In addition, the defect in transmission was correlated with a lack of virus retention by *X. index*, although virus acquisition by nematodes was not affected. Therefore, Gly^297^ is a *bona fide* determinant of GFLV transmission efficiency.

The high-resolution structure of GFLV reveals an overall organization well in agreement with its classification in the order *Picornavirales* within the picorna-like super family [44], [45]. The GFLV capsid consists of 60 subunits, each containing three distantly related jellyroll domains that may have arisen by triplication of a single copy present in some ancestor virus and subsequent divergent evolution [31], [46]. The high degree of similarity of the 3D structures of GFLV and TRSV (**Figure S3, Table S1**) is in agreement with their classification in the same genus [47]. Yet, the superposition of their capsid proteins is not perfect. This is mainly due to small differences in the orientation of subunits within particles and the length of surface loops, *e.g*. GH loop in the B-domain. These differences certainly explain why classical molecular replacement using homology models was unsuccessful. Indeed, our initial 3D model of GFLV [32] resembled more TSRV from which it was derived than the actual crystal structure (**Figure S3C**). In contrast, the 16.5 Å cryoEM map of GFLV (**Figure S2**) rapidly led to an unambiguous solution. Overall, the resulting structures of GFLV-F13 and GFLV-TD have identical architectures although they were determined in different crystalline packings [36]. These findings indicate that particles are quite rigid and, more importantly, that the differential ability to be transmitted is not due to a conformational modification but rather to an alteration of the physical-chemical properties of their outer surface.

Single point mutations detrimental to virus transmission often affect highly conserved residues. For instance, single mutations in the conserved HI loop of CMV either reduce or abolish aphid transmission [18]. Also, single mutations in the conserved PTK motif of ZYMV HC-Pro [14] or in the DAG motif of TYMV CP [48] hinder aphid transmission of potyviruses. In GFLV, Gly^297^ is a highly conserved amino acid of the GH loop and our structure shows that it is very accessible to the solvent. Out of the 238 GFLV CP sequences available to date in GenBank, only three allelic variants exist at this position: Ser^297^ (accession number 38604190), Asn^297^ (accession number 86450421) and Asp^297^ (reported for GFLV isolate CACSB5 from California with accession number 299118269 [49] and this work). The transmissibility of the Ser^297^ and Asn^297^ allelic variants and CACSB5 isolate is unknown. Here we show that the Gly^297^Asp strongly affects transmission. We also found that the Gly^297^Ala single mutant (GFLV-G^297^A) is still transmitted by *X. index,* although Ala is the most frequent residue at position 297 in the CP of ArMV strains. Altogether, this indicates that the nature of the side chain of residue occupying the position 297 is decisive for vector recognition.

Since the same structure is observed in GFLV-TD and GFLV-F13, a conformational effect of the Gly_297_Asp mutation cannot account for the deficiency in transmission of GFLV-TD. However, the Asp^297^ side chain could create a steric hindrance and thereby interfere with proper recognition of a ligand within the nematode feeding apparatus. A more likely scenario is that Asp^297^ perturbs the electrostatic potential at the surface of the virions and their solvation shell via the addition of 60 negative charges in GFLV-TD. A striking consequence of this alteration is a 2.5-fold increase of the solubility of GFLV-TD with respect to that of wild-type GFLV-F13. Another one is the different crystal packing [36]. In the same way, alteration of the electrostatic potential may also impair the binding and retention of GFLV inside the nematode feeding apparatus, thereby reducing its transmissibility. Future work will clarify which hypothesis, electrostatic potential or steric hindrance, contributes most to the loss of transmission of GFLV-TD.

Our results show that Gly^297^ and region R2 are transmission determinants but they cannot alone explain the strict transmission specificity between GFLV and *X. index*. Thus, these residues may be part of an ensemble of surface residues with ligand binding properties. In view of our structural data, it appears that they are located at the edge of a pocket near the 3 fold axis whose walls are formed essentially by the GH, BC and C′C″ loops within the B-domain. This pocket is remarkable in several respects. First, it is positively charged whereas most of the GFLV outer surface is negatively charged (**Figure 5A**). Second, all three loops contain residues that are protruding from the capsid outer surface (**Figure 3** **, Figure S5**) and are therefore likely to be recognized by compounds of the nematode feeding apparatus. Finally, these three loops were previously identified for their possible involvement in nematode transmission and the function of region R2 encompassing the BC loop was experimentally proven [32]. For all these reasons, we suggest that this pocket may constitute a ligand recognition site with critical function in GFLV transmission by *X. index*. We also note that its topology resembles the receptor-binding site of other picorna-like viruses, in particular the heparin binding site of *Foot-and-mouth disease virus* (FMDV) where the pocket occupies a similar position within the icosahedral asymmetric unit (**Figure S7**) and contains important polar and positively charged residues with ligand binding properties [50], [51]. Whether the occurrence of negatively charged residues in the pocket is detrimental for GFLV transmission by its vector needs to be confirmed. Indeed, so far only two mutants, namely Phe_188_Glu (*i.e.* the first residue of R2, [32]) and Gly_297_Asp (described as GFLV-TD in this work) have been identified in which an alteration of the net electrostatic charge inside the putative ligand-binding pocket was correlated to a defect in virus transmission.

This work provides a new framework for further analyses aiming at precisely defining the structure and charge properties of the binding pocket and of its importance for GFLV transmission by nematodes. The knowledge of the underlying molecular mechanisms is a prerequisite for the identification of a ligand within the nematode feeding apparatus and the subsequent development of novel strategies to control virus propagation in vineyards.

In conclusion, effective virus transmission from host to host relies on a specific interaction with a vector. Here, we have identified structural features involved in such interaction on the surface of a 30 nm icosahedral nepovirus. We showed that a single mutation (Gly_297_Asp) in the GH loop within the CP B domain is sufficient to diminish GFLV transmission by its ectoparasitic nematode vector *X. index*. In the absence of any detectable difference in the resolved 3D structures of the wild-type virus and a transmission deficient mutant, we come to the conclusion that the introduction of a negative charge at a precise position in each of the 60 protein subunits of the capsid is sufficient to diminish virus retention inside the nematode's feeding apparatus and thereby hinder virus transmission. We have also delimited a positively charged pocket formed at the surface of the protein capsid which may constitute a binding site for the vector. These findings open new perspectives for the mapping of the ligand recognition site on the virus and the identification of a viral receptor or ligand in the nematode. Providing deeper insights into virus-vector interactions at the atomic level will help understand the origin of the specificity of virus-vector interactions and facilitate the implementation of new strategies to break the viral cycle.

## Materials and Methods

### Virus strains and plant infection with viral transcripts

GFLV and ArMV strains were isolated from naturally infected grapevines and propagated in the systemic host *C. quinoa*. Full-length cDNA clones of GFLV-F13 RNA1 and RNA2 are available [35]. They were used to produce RNA molecules by *in vitro* transcription as described previously [52]. Transcripts of either wild-type GFLV RNA1 and RNA2 or GFLV RNA1 and mutated RNA2 were mechanically inoculated to *C. quinoa* [35]. Virus infection was assessed in uninoculated apical leaves of *C. quinoa* plants 2 to 3 weeks post-inoculation by double-antibody sandwich (DAS)-enzyme-linked immunosorbent assay (ELISA) with specific γ-globulins to GFLV and ArMV. Samples were considered positive if their optical density (OD_405nm_) readings were at least three times those of healthy controls after 120 min of substrate hydrolysis.

### GFLV purification and crystallization

Viral particles were purified mainly as described in [53] with one additional 60 to 10% (m/v) sucrose gradient that was performed at 210,000× *g* in SW41 rotor (Beckman) for 2.5 h. Purified virions were resuspended in sterile water and filtered through a 0.22 µm pore-size Ultrafree-MC membrane (Millex) prior to storage at 4°C. Crystallization by vapor diffusion at 20°C in sub-microliter sitting drops and structural analyses were performed as described [36].

### Mutagenesis of GFLV RNA2

Plasmid pVec*_Acc_*_65I_2ABC, carrying a full-length cDNA copy of GFLV RNA2 was used as template for the production of chimeric CP genes harboring a mutated amino acid in position 297 by PCR site directed mutagenesis overlap extension mutagenesis [32]. Plasmid pVec*_Acc_*_65I_2ABC_G2_ is a derivative of pVec*_Acc_*_65I_2ABC carrying the CP region R2 in position nts 2,609–2,640 (nucleotide positions are given according to the GFLV-F13 RNA2 sequence, GenBank accession no. NC_003623) [32]. Residue 297 (corresponding to codon nts 2,936–2,938) was mutated into an aspartic acid, using pVec*_Acc_*_65I_2ABC as template, the mutagenic primer pair mutDF/mutDR and the external primer pair 18/36 (**Table S2**). Mutagenic PCR-amplified products were digested with *Acc65I* (nts 2,678–2683) and *BglII* (nts 3,055–3,060) and cloned into the corresponding sites in pVec*_Acc_*_65I_2ABC to yield pVec*_Acc_*_65I_2ABC_G297D_. Residue 297 was mutated into Alanine with the mutagenic primers mutAF/mutAR and the external primers 18/36 (**Table S2**); PCR-amplified products were digested with *Acc65I* and *BglII*, and cloned into the corresponding sites in pVec*_Acc_*_65I_2ABC and pVec*_Acc_*_65I_2ABC_G2_ to yield pVec*_Acc_*_65I_2ABC_G297A_ and [54] pVec*_Acc_*_65I_2ABC_G2-G297A_, respectively. Each PCR reaction was carried out as described [32]. For simplicity, transcripts and mutant viruses derived from these constructs were referred to as GFLV-G_297_D (plasmid pVec*_Acc_*_65I_2ABC_G297D_), GFLV-G_297_A (plasmid pVec*_Acc_*_65I_2ABC_G297A_), and GFLV-R2G_297_A (plasmid pVec*_Acc_*_65I_2ABC_G2-G297A_). The integrity of all GFLV RNA2 clones was verified by DNA sequencing.

### Nematode transmission tests and detection of GFLV and ArMV in nematodes

Nematode transmission assays were performed in two steps of 4 weeks each, the acquisition access period and the inoculation access period [30]. *C. quinoa* and *Nicotiana benthamiana* were used as source and bait plants for transmission assays with *X. diversicaudatum* and *X index,* respectively. Transmission tests were performed using 200 nematodes per plant. The presence of GFLV and ArMV was verified in total RNA extracts from nematodes by reverse-transcription (RT)-polymerase-chain reaction (PCR) as described [30].

### Characterization of GFLV RNA2 progeny

The progeny of GFLV RNA2 CP sequence was characterized in infected plants by immuno-capture (IC)-RT-PCR and sequencing as described in [29], except that two cDNA fragments were amplified with primer pairs 397/227 and 115/18 (see **Table S2**). Sequences were analyzed with ContigExpress (Vector NTI Software, InforMax).

### Cryo-electron microscopy 3D reconstruction

Purified GFLV particles were applied to a quantifoil R 2/2 carbon grid (Quantifoil Micro Tools GmbH, Germany), blotted by filter paper, and flash-frozen in liquid ethane to make a vitreous-ice embedded sample. Electron micrographs were recorded under low-dose conditions at liquid-N2 temperature with a JEOL 2010 operating at 200 kV microscope. Micrographs collected at X 50,000 magnification with a defocus range of 1.3–2.5 µm were digitized on a Nikon Coolscan 9000 ED with a step size of 10 µm. The images were coarsened by a factor of 2, resulting in a pixel size corresponding to 4 Å at the specimen level. The semi-automatic X3D program (J.F. Conway) was used for picking particles. The defocus value was estimated for each micrograph using CTFFIND3 [55], and phases flipped using CTFMIX [56]. Particle origins and orientations were determined and refined using the model-based orientation determination method [57]. The GFLV reconstruction was determined using as starting model the 3D reconstruction of TRSV filtered at 40 Å resolution. The density map was calculated by Fourier-Bessel formalism as described [57], and implemented in the EM3DR program. Resolution was estimated using the Fourier shell correlation (FSC) criterion with a cutting level of 0.5 [58]. The final density map computed at 16.5 Å resolution includes 2,424 particles extracted from 8 micrographs.

### X-ray structure determination and analysis

X-ray diffraction data from GFLV-F13 and GFLV-TD were collected on crystal-cooled samples (**Table 1**) at FIP-BM30 beamline (ESRF, Grenoble, France) and at X06DA beamline (SLS, Villingen, Switzerland). They were reduced using the *XDS* package [59].

Diffraction data were phased by molecular replacement using *AMoRe* [60] followed by non-crystallographic symmetry (NCS) averaging and solvent flattening using *RAVE* [61], [62]. Attempts to phase data using TRSV-based homology models generated by Modeller [63] were not successful. In contrast, the 3D EM reconstruction led to a clear molecular replacement solution with cubic data in the 30-15 Å resolution range. The orientations of viral particles within the cubic crystal were identified by inspection of the self-rotation function calculated at the highest resolution available (4.5 Å for GFLV-F13 and 2.85 Å for GFLV-TD). Self-rotations corresponding to four differently oriented icosahedral particles were found. Calculation of the translation-function using the correctly oriented 3D EM model showed that four icosahedral particles were present in the unit cell, each sharing one of its 3-fold axis with the crystal. The molecular replacement solutions defined the molecular boundaries (masks) of the particles within the cubic crystals. Based on the icosahedral symmetry of the 3D EM model, the rigid-body operators relating equivalent regions within the molecular boundaries were defined (20 NCS x 3 crystallographic transformations). An iterative procedure of phase extension from 16.5 Å to the maximum available resolution was then carried out by using density modification techniques, including NCS map averaging, solvent flattening and intermediate steps where the molecular masks and the NCS operators were refined.

The incorporation of high-resolution data finally converged to an experimental map at 2.85 Å which allowed the rapid rebuilding of GFLV subunit from homology models. The atomic model of GFLV-TD was refined with *PHENIX* [64] with cubic data reprocessed at 2.7 Å resolution. NCS constrains were applied to the ensemble of monomers in the asymmetric unit except three regions which changed conformation due to packing contacts (Tyr 9, loops 15–19 and 259–265). Water molecules were added after convergence of capsid refinement. Strong peaks in the difference map were examined in *Coot* [65] to identify 28 solvent molecules around one monomer A. They were then transferred by symmetry to subunits B-T and a total of 556 solvent sites were assigned in the final model. Strong density peaks were also observed on the 5-fold axes of the capsid indicating the presence of a large ion, possibly a phosphate. A ring of solvent molecules bridging the ion to the CP monomers was clearly seen in 2 out of 4 pentamers of the cubic asymmetric unit. However, this ion could not be explicitly identify (no exploitable anomalous signal) and was not included in the model. The structure of the GFLV-F13 particle was solved by MR using the GFLV-TD model and was refined at 3 Å resolution. No solvent molecule was included, since it was not possible at this resolution to describe a common hydration pattern for the 60 viral subunits in the asymmetric unit. The stereochemical quality (**Table 1**) of final models was assessed with *Coot* and *Procheck* [65] and all residues were in the allowed regions of the Ramachandran plot. The totality of the CP amino acids (504 residues per subunit) was observed in both GFLV-F13 and -TD GFLV structures. Atomic coordinates have been deposited in the Protein Databank (GFLV-TD: pdb ID 2Y26; GFLV-F13: 2Y7T, 2Y7U, 2Y7V).

GFLV structures were compared with *lsqman* [61]. R.m.s.d. on Cα positions were calculated for each pairwise superimposition of CPs observed in the cubic (GFLV-TD) and in the monoclinic (GFLV-F13) asymmetric units. Average r.m.s.d. were derived from the former analysis and are reported in **Table S1**, as well as the r.m.s.d of GFLV CP vs TRSV CP and GFLV CP model based on TRSV.[61]. Solvent accessible surface was calculated with a probe radius of 1.4 Å, with the program *MSMS* [66]. The analysis of the surface potential was performed with *APBS* [57], [67]. Figures were prepared using *PyMol* (http://www.pymol.org/) and *Chimera* [67].

## Supporting Information

Figure S1 Nematode transmission assays. Prior to the transmission assays, the infectious status of all source plants - C. quinoa or N. benthamiana - was verified by DAS-ELISA using specific GFLV and ArMV antibodies. 200 aviruliferous nematodes were allowed to feed on the roots of a virus source plant for a four-week acquisition access period (AAP). Then, nematodes were exposed to the roots of healthy bait plants for a four-week inoculation access period (IAP). The successful transmission of viruses by nematodes was verified in the roots of each bait plant by DAS-ELISA using specific GFLV and ArMV antibodies.
(TIF)

Figure S2 Isosurface representation of the GFLV-TD reconstruction at 16.5 Å resolution obtained after cryoelectron microscopy. The symmetry axes are marked with a pentagon (five-fold), triangle (three-fold) and bar (two-fold).
(TIF)

Figure S3 Structural similarity of GFLV and TRSV. This stereoview shows a superposition of the GFLV-F13 CP (Cα trace representation) and that of GFLV-TD (A), TRSV (B) and GFLV homology model derived from the TRSV crystal structure (C), respectively. The C, B and A domains in GFLV are shown in green, red and blue, respectively. The subunit is viewed from the outside of the capsid. Other structures are depicted in grey. The position of GFLV residue 297 in the GH loop is indicated. The structures were superimposed using *lsqman* [61]. Corresponding r.m.s.d. values are listed in Table S1.
(TIF)

Figure S4 Capsid protein contacts on 3-fold and 2-fold axes. (A) The black line delineates one CP position. The figure indicates the contact between different domains on the 3-fold and 2-fold axes. The grey pentagon, triangle and oval symbolize the icosahedral 5-fold, 3-fold and 2-fold symmetry axes, respectively. Domains of the same CP are labelled with the same number. The A domains are exclusively clustered around the 5-fold axis. (B) Six β-barrels from B and C domains belonging to different CPs interact around the 3-fold axis. (C) Two B and C domains from four CPs interact on the 2-fold axis.
(TIF)

Figure S5 Position of nematode transmission determinants on GFLV capsid surface. (A) This close-up view of the capsid reveals that residue Gly^297^ (purple) and region R2 comprising residues 188 to 198 (orange) are facing the outer surface of the capsid. (B) In this stereoview, a single CP is seen from the outside of the capsid with C, B, and A domains colored blue, red and green, respectively. The distances in Å between Gly^297^ (purple) and residues from region R2 (orange) are indicated.
(TIF)

Figure S6 Virus detection in *Xiphinema* species at the end of the acquisition access period (AAP). Nematodes exposed to source plants infected with GFLV-F13 (2), GFLV-G_297_D (3), GFLV-G_297_A (4), GFLV-R2G_297_A (5), or ArMV (6) and mock inoculated plants (1 and 6) were randomly selected and characterized by RT-PCR. The amplification of specific DNA products confirmed that the nematodes had ingested all types of viruses during AAP.
(TIF)

Figure S7 Comparison between the GFLV putative ligand-binding pocket and the FMDV heparin sulphate binding site. (A) GFLV CP is seen from the outside of the capsid with the C, B, and A domains colored as in Figure 2, and the putative ligand binding pocket in white. (B) FMDV viral proteins VP1, VP2, VP3 and VP4 (pdb ID, 1QQP) are colored in blue, red, green and yellow, respectively. Residues involved in heparin sulphate binding [50], [51] appear in white. Grey pentagon, triangle and oval symbolize the icosahedral 5-fold, 3-fold and 2-fold symmetry axes, respectively.
(TIF)

Table S1 Comparison of capsid proteins and of CP domains A, B, and C.
(DOC)

Table S2 Primers used to produce gene 2C^CP^ with mutated residues and to characterize GFLV RNA2 progeny.
(DOC)
