# Correction: Structural Insights into Viral Determinants of Nematode Mediated *Grapevine fanleaf virus* Transmission

**DOI:** 10.1371/journal.ppat.1006268

**Published:** 2017-03-15

**Authors:** Pascale Schellenberger, Claude Sauter, Bernard Lorber, Patrick Bron, Stefano Trapani, Marc Bergdoll, Aurélie Marmonier, Corinne Schmitt-Keichinger, Olivier Lemaire, Gérard Demangeat, Christophe Ritzenthaler

The authors would like to correct Figure 1C, as errors were made in the preparation of this figure for publication. In the original Figure 1C, in the AAP panel, lanes 1 and 2 were duplicated and in the IAP panel, lanes 3 and 4 were duplicated. The authors were unable to locate the original data, however, the experiment reported in the original Figure 1C was repeated twice with identical scientific conclusions. The results of the repeated experiment are presented in the corrected Fig 1C. The new experiments used to generate the data was comprised of three transmission tests and was not specifically dedicated to the sole GFLV-TD and GFLV-G297D mutants and include other unpublished GFLV mutants. Therefore, to assemble the new figure, the authors assembled parts from the larger experiment and therefore have included some additional lanes in the corrected Fig 1C. The corrected Fig 1C and corrected figure legend are presented here. In addition, the authors have provided the raw uncropped blots used to assemble the corrected Fig 1C as Supporting Information. The authors confirm that these changes do not alter their findings. The authors apologize for any concern this may have caused.

**Fig 1:**
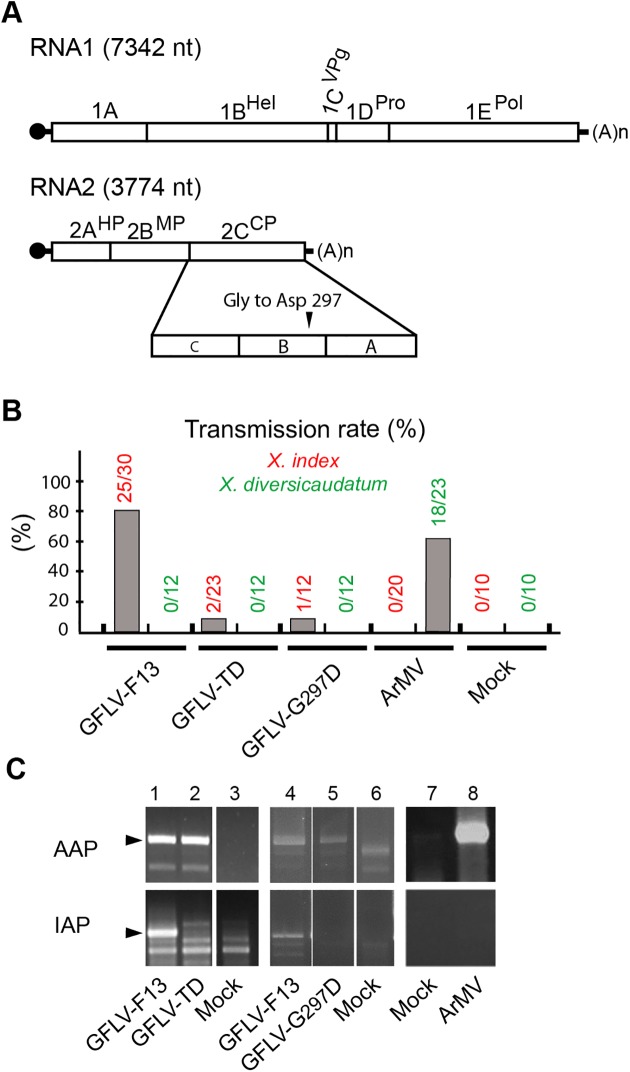
Involvement of capsid protein residue 297 in nematode transmission. **(A)** Genomic organization of GFLV. The 5′ and 3′ untranslated regions are denoted by single lines and the VPg is represented by a black circle. Polyproteins encoded by RNA1 and RNA2 are cleaved in five (1A–1E) and three (2A–2C) final maturation products (open boxes), respectively. 1B, helicase (Hel); 1C, viral protein genome-linked (VPg); 1D, protease (Pro); 1E, RNA-dependent RNA polymerase (Pol); 2A, homing protein (HP); 2B, movement protein (MP) and 2C, coat protein (CP). As indicated, the CP is composed of three domains called C, B, and A. In the variant GFLV-TD, the CP residue Gly at position 297 is replaced by Asp. **(B)** Transmission of wild type GFLV-F13, GFLV-TD and GFLV-G297D (the two latter with a Gly297 to Asp297 substitution) and wild type ArMV by *X*. *index* and *X*. *diversicaudatum*. Transmission rates are expressed as the percentage of ELISA-positive plants. **(C)** The virus detection in *X*. *index* at the end of the AAP and the IAP showed that the mutated viruses and ArMV were ingested but only poorly (GFLV-TD) or not retained by nematodes (GFLV-G297D, ArMV). Thirty nematode specimens exposed to source plants infected with GFLV-F13 (lane 1, 4), GFLV-TD (lane 2), GFLV-G297D (lane 5), ArMV (lane 8), or mock inoculated plants (lanes 3, 6 and 7) were randomly collected and tested by RT-PCR with GFLV- (lanes 1–6) or ArMV- (lanes 7 and 8) specific primers. DNA products were analyzed by electrophoresis on 1.5% agarose gels. Positions of the expected specific DNA fragments are indicated by arrows.

## Supporting information

S1 Fig Raw uncropped blots for Fig 1C. The nematode transmission tests were performed in a greenhouse using aviruliferous *X*. *index* nematodes isolated from rearings established on fig plants (*Ficus carica*). Transmission test is a two-step procedure: an acquisition access period (AAP) and an inoculation access period (IAP). About 300 nematodes were given access to the roots of an infected *Nicotiana benthamiana* source plant. After an AAP of 6 weeks, infected source plant was replaced by a healthy *N*. *benthamiana* bait plant and grown in greenhouse for an IAP of 6 weeks. At the end of each step, nematodes were randomly collected. The presence of virus was verified in nematodes by reverse transcription-PCR (RT-PCR) with GFLV or ArMV-specific primers. Panels 1 to 3 below correspond to AAP analyses. Panel 4 to 6 below correspond to IAP analyses. Red boxes indicate the cropped area used to assemble the corrected Fig 1C with lanes indicated in red below the panels.
(PPTX)
