# Supplementary figures and images for: Structural Insights into Viral Determinants of Nematode Mediated Grapevine fanleaf virus Transmission
Source: PLoS Pathog. 2011 May 19;7(5):e1002034. doi: 10.1371/journal.ppat.1002034 (PMC3098200; doi:10.1371/journal.ppat.1002034)

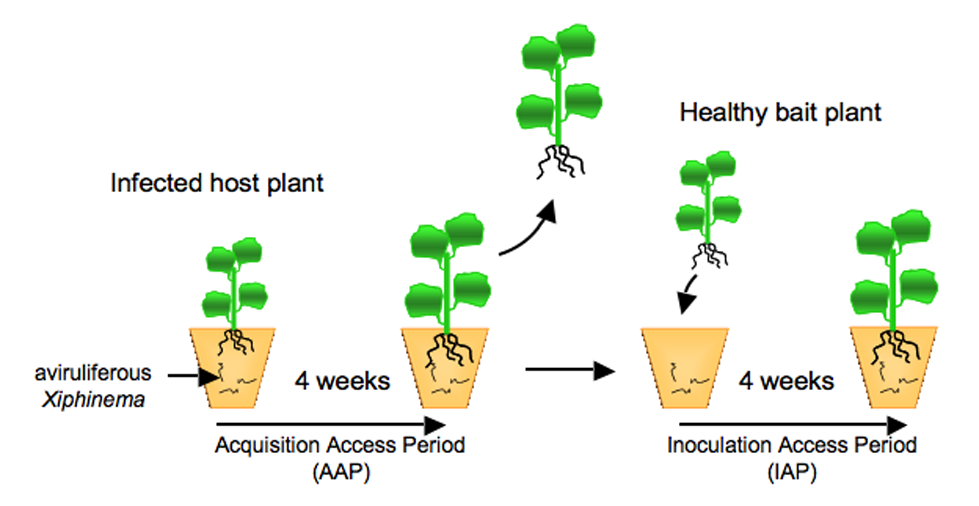

Supplement: Figure S1 — Nematode transmission assays. Prior to the transmission assays, the infectious status of all source plants - C. quinoa or N. benthamiana - was verified by DAS-ELISA using specific GFLV and ArMV antibodies. 200 aviruliferous nematodes were allowed to feed on the roots of a virus source plant for a four-week acquisition access period (AAP). Then, nematodes were exposed to the roots of healthy bait plants for a four-week inoculation access period (IAP). The successful transmission of viruses by nematodes was verified in the roots of each bait plant by DAS-ELISA using specific GFLV and ArMV antibodies. (TIF) [file ppat.1002034.s001.tif]

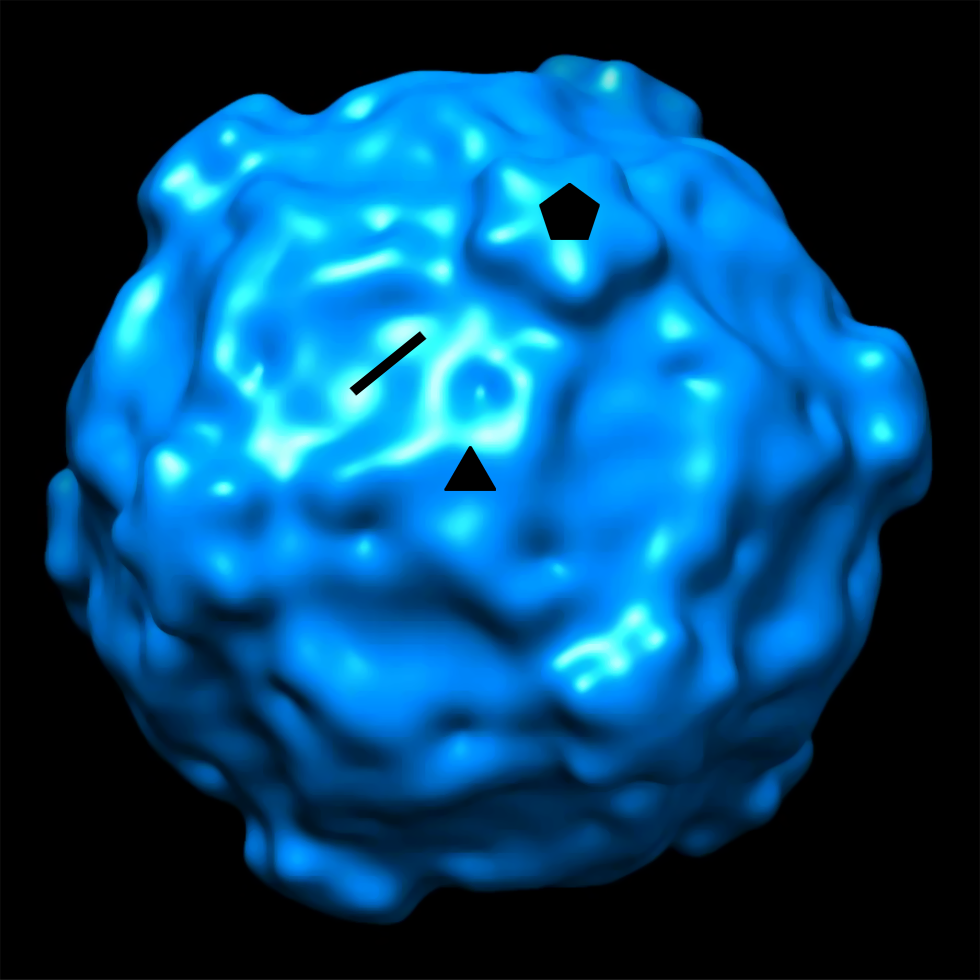

Supplement: Figure S2 — Isosurface representation of the GFLV-TD reconstruction at 16.5 Å resolution obtained after cryoelectron microscopy. The symmetry axes are marked with a pentagon (five-fold), triangle (three-fold) and bar (two-fold). (TIF) [file ppat.1002034.s002.tif]

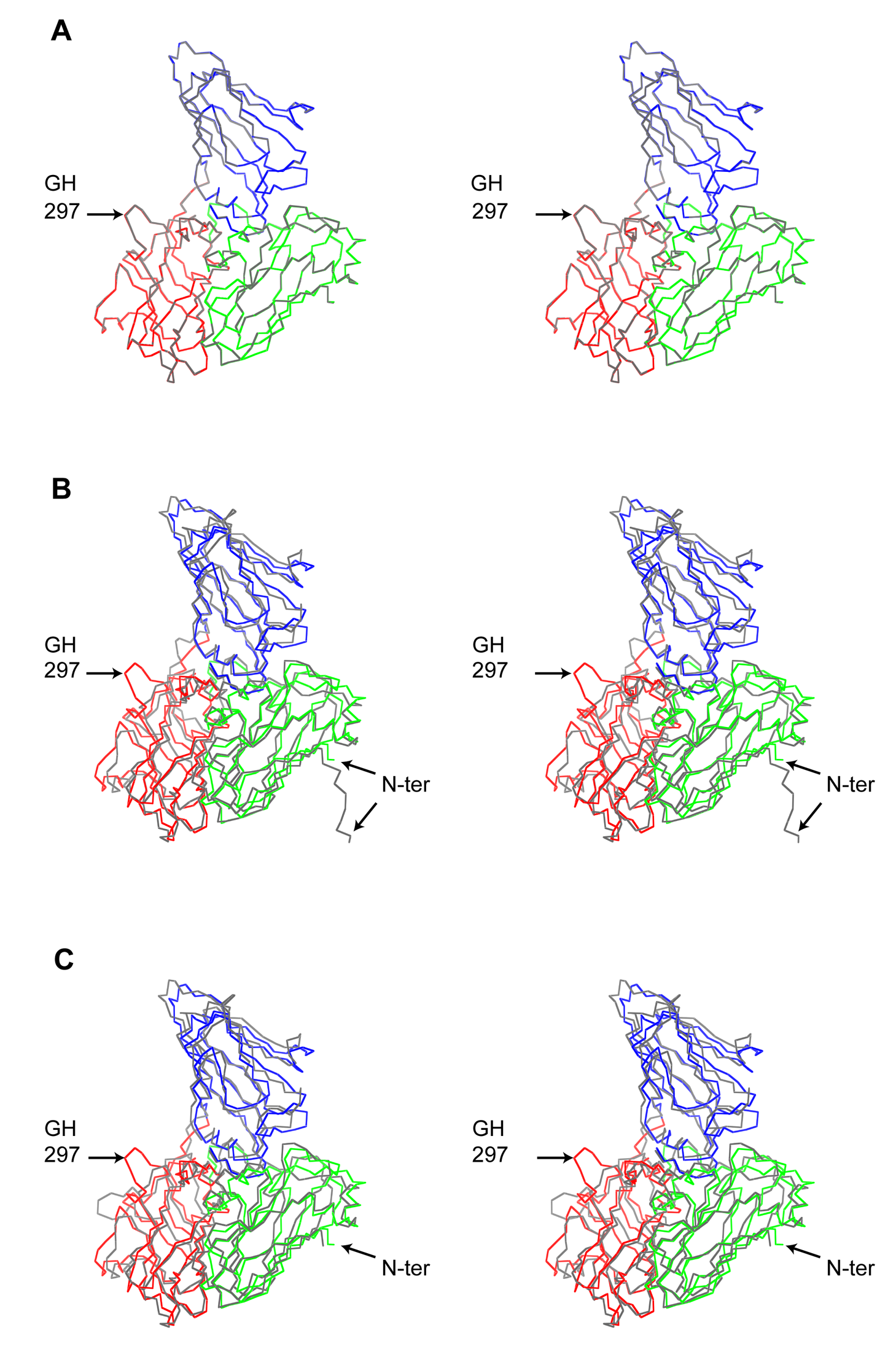

Supplement: Figure S3 — Structural similarity of GFLV and TRSV. This stereoview shows a superposition of the GFLV-F13 CP (Cα trace representation) and that of GFLV-TD (A), TRSV (B) and GFLV homology model derived from the TRSV crystal structure (C), respectively. The C, B and A domains in GFLV are shown in green, red and blue, respectively. The subunit is viewed from the outside of the capsid. Other structures are depicted in grey. The position of GFLV residue 297 in the GH loop is indicated. The structures were superimposed using lsqman [61]. Corresponding r.m.s.d. values are listed in Table S1. (TIF) [file ppat.1002034.s003.tif]

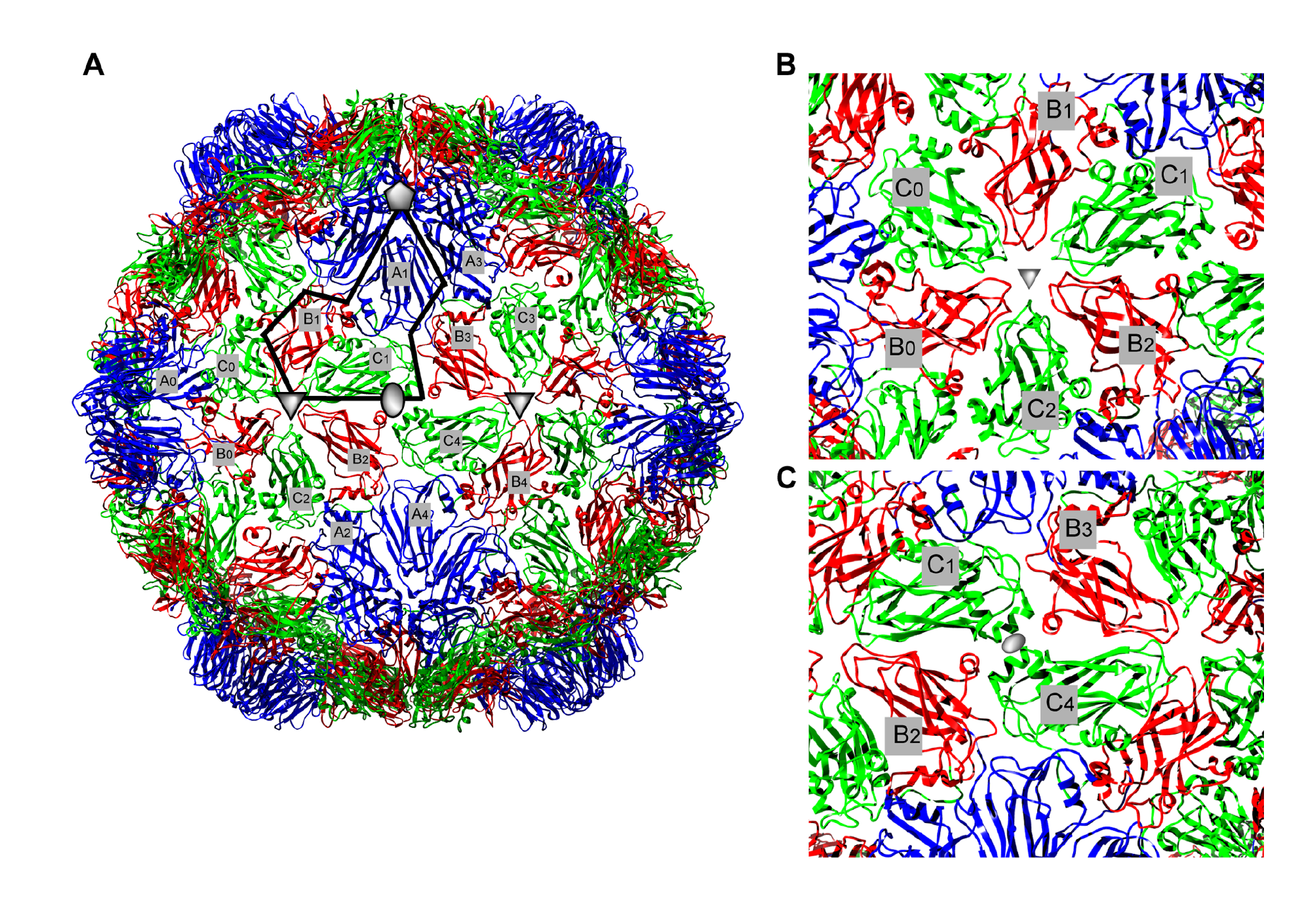

Supplement: Figure S4 — Capsid protein contacts on 3-fold and 2-fold axes. (A) The black line delineates one CP position. The figure indicates the contact between different domains on the 3-fold and 2-fold axes. The grey pentagon, triangle and oval symbolize the icosahedral 5-fold, 3-fold and 2-fold symmetry axes, respectively. Domains of the same CP are labelled with the same number. The A domains are exclusively clustered around the 5-fold axis. (B) Six β-barrels from B and C domains belonging to different CPs interact around the 3-fold axis. (C) Two B and C domains from four CPs interact on the 2-fold axis. (TIF) [file ppat.1002034.s004.tif]

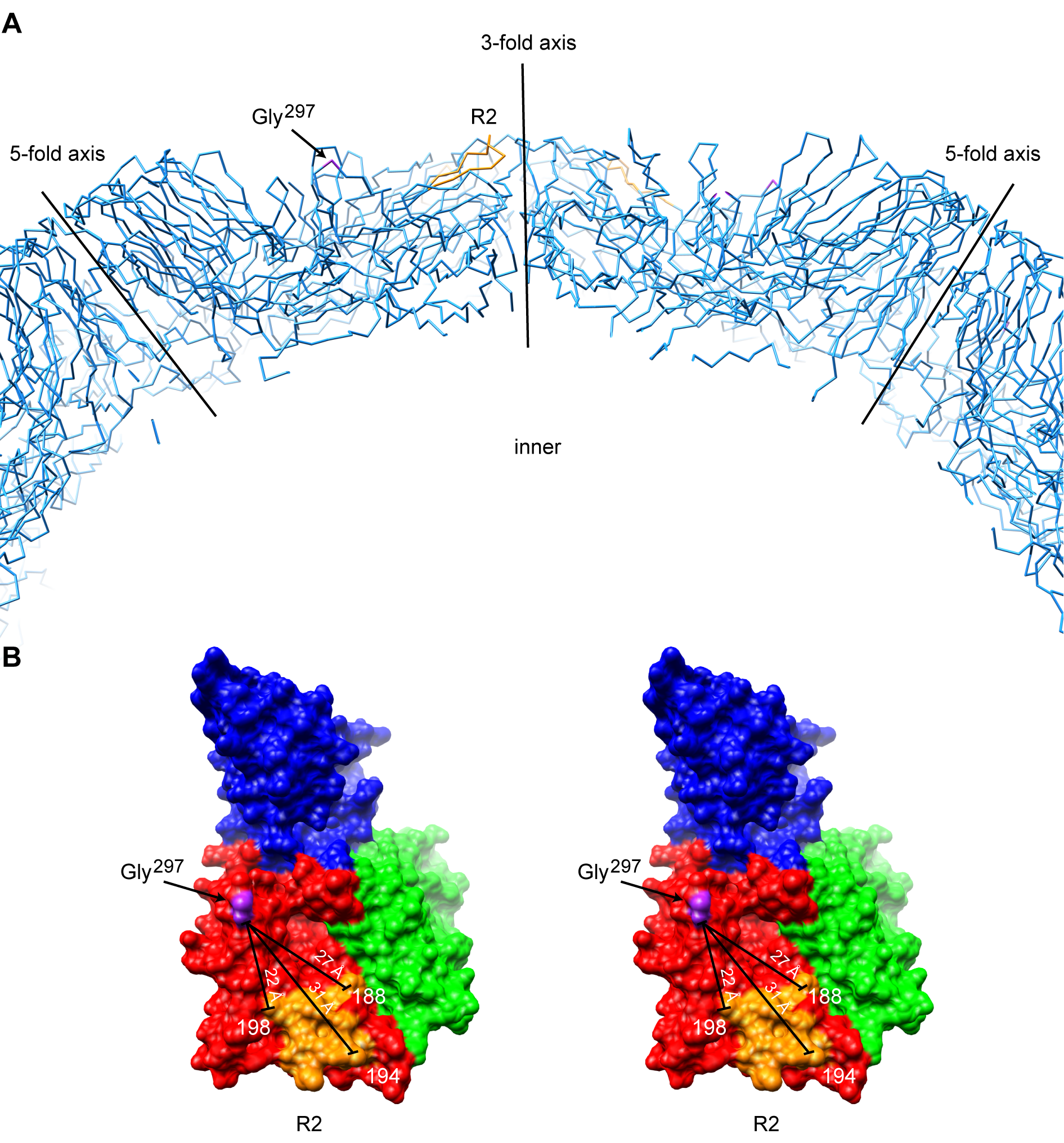

Supplement: Figure S5 — Position of nematode transmission determinants on GFLV capsid surface. (A) This close-up view of the capsid reveals that residue Gly297 (purple) and region R2 comprising residues 188 to 198 (orange) are facing the outer surface of the capsid. (B) In this stereoview, a single CP is seen from the outside of the capsid with C, B, and A domains colored blue, red and green, respectively. The distances in Å between Gly297 (purple) and residues from region R2 (orange) are indicated. (TIF) [file ppat.1002034.s005.tif]

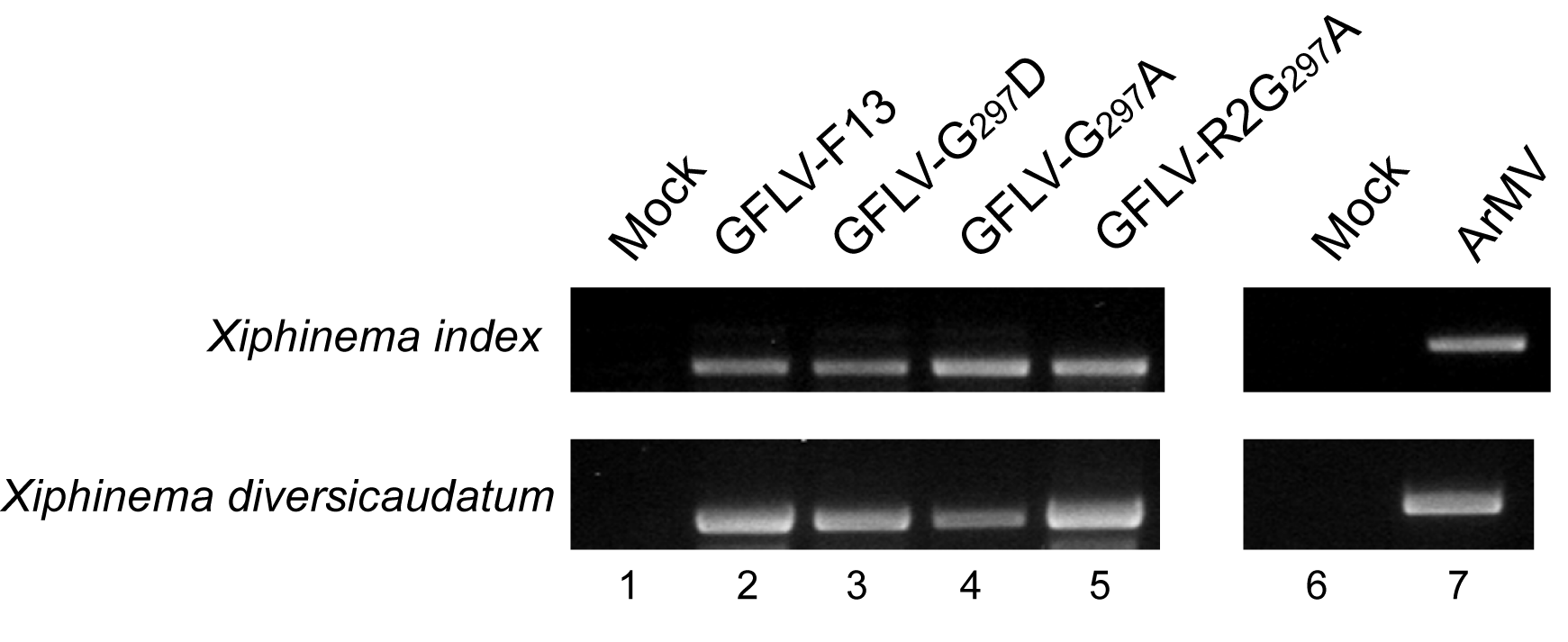

Supplement: Figure S6 — Virus detection in Xiphinema species at the end of the acquisition access period (AAP). Nematodes exposed to source plants infected with GFLV-F13 (2), GFLV-G297D (3), GFLV-G297A (4), GFLV-R2G297A (5), or ArMV (6) and mock inoculated plants (1 and 6) were randomly selected and characterized by RT-PCR. The amplification of specific DNA products confirmed that the nematodes had ingested all types of viruses during AAP. (TIF) [file ppat.1002034.s006.tif]

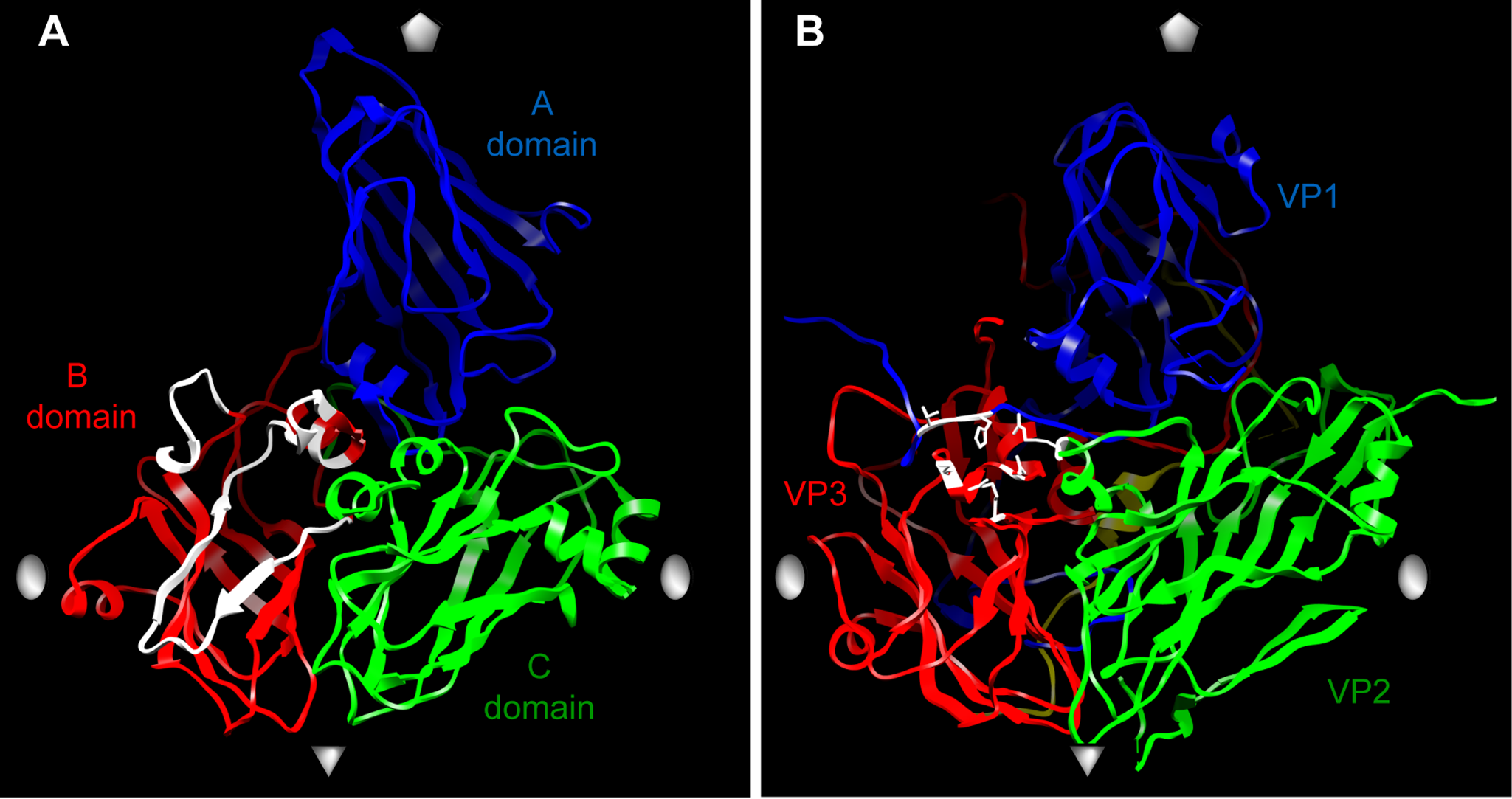

Supplement: Figure S7 — Comparison between the GFLV putative ligand-binding pocket and the FMDV heparin sulphate binding site. (A) GFLV CP is seen from the outside of the capsid with the C, B, and A domains colored as in Figure 2, and the putative ligand binding pocket in white. (B) FMDV viral proteins VP1, VP2, VP3 and VP4 (pdb ID, 1QQP) are colored in blue, red, green and yellow, respectively. Residues involved in heparin sulphate binding [50], [51] appear in white. Grey pentagon, triangle and oval symbolize the icosahedral 5-fold, 3-fold and 2-fold symmetry axes, respectively. (TIF) [file ppat.1002034.s007.tif]
