# Supplementary material for: Structural Insights into Viral Determinants of Nematode Mediated Grapevine fanleaf virus Transmission
Source: PLoS Pathog. 2011 May 19;7(5):e1002034. doi: 10.1371/journal.ppat.1002034 (PMC3098200; doi:10.1371/journal.ppat.1002034)
Supplement: Table S1 — Comparison of capsid proteins and of CP domains A, B, and C. (DOC) [file ppat.1002034.s008.doc]

**Table S1**

| Structural comparison | Superimposed pair | r.m.s.d. (Å)^a^ | No. of matched residues |
| --- | --- | --- | --- |
| GFLV-TD (20 CPs) | CP CP | 0.07±0.01^b^ | 504 |
| GFLV-F13 (60 CPs) | CP CP | 0.10±0.02^c^ | 504 |
| GFLV-F13 *vs* GFLV-TD | CP CP | 0.12±0.02^d^ | 504 |
| GFLV-F13 *vs* TRSV | CP CP  A A  B B  C C | 1.7  1.42  1.30  1.19 | 444  143  130  145 |
| GFLV-F13 vs homology model | CP CP | 1.62^e^ | 429 |

^a^Root mean square distances calculated on Cα positions

^b^Average value on 190 pairwise comparisons

^c^Average value on 1770 pairwise comparisons

^d^Average value on 1200 pairwise comparisons

^e^e-Value for Chain A of GFLV-F13
