# Supplementary material for: Structural Insights into Viral Determinants of Nematode Mediated Grapevine fanleaf virus Transmission
Source: PLoS Pathog. 2011 May 19;7(5):e1002034. doi: 10.1371/journal.ppat.1002034 (PMC3098200; doi:10.1371/journal.ppat.1002034)
Supplement: Table S2 — Primers used to produce gene 2CCP with mutated residues and to characterize GFLV RNA2 progeny. (DOC) [file ppat.1002034.s009.doc]

Table S2

| Name | Direction-position^a^ | Sequence^b^ |
| --- | --- | --- |
| mutDF | F-2918 | CCTGCCAGATTGCTTGCTG**A**TCAAAGTCAGAGAGACATGAGC |
| mutDR | R-2959 | GCTCATGTCTCTCTGACTT**T**GATCAGCAAGCAATCTGGCAGG |
| mutAF | F-2918 | CCTGCCAGATTGCTTGCTG**C**TCAAAGTCAGAGAGACATGAGC |
| mutAR | R-2959 | GCTCATGTCTCTCTGACTT**T**GAGCAGCAAGCAATCTGGCAGG |
| 36 | F-2522 | GCCACTTCTTTCCTTGGGAAACCAA |
| 18 | R-3103 | ATCCACCCATACGAAATAGTC |
| 227 | F-2862 | ATGTGGAAGAGGACGGAAGT |
| 397 | R-3722 | GGCAAGTGTGTCCAAAGGAC |
| 115 | F-1977 | CTGTGAGGATTGATAGAAACG |

^a^Primer positions are given as the position of the 5’ first nucleotide according to the GFLV-F13 RNA2 sequence (NC_003623). F = forward and R = reverse orientation.

^b^Mutagenic nucleotides are highlighted in grey.
