# Supplementary figures and images for: Correction: Structural Insights into Viral Determinants of Nematode Mediated Grapevine fanleaf virus Transmission
Source: PLoS Pathog. 2017 Mar 15;13(3):e1006268. doi: 10.1371/journal.ppat.1006268 (PMC5352166; doi:10.1371/journal.ppat.1006268)

## Slide 1
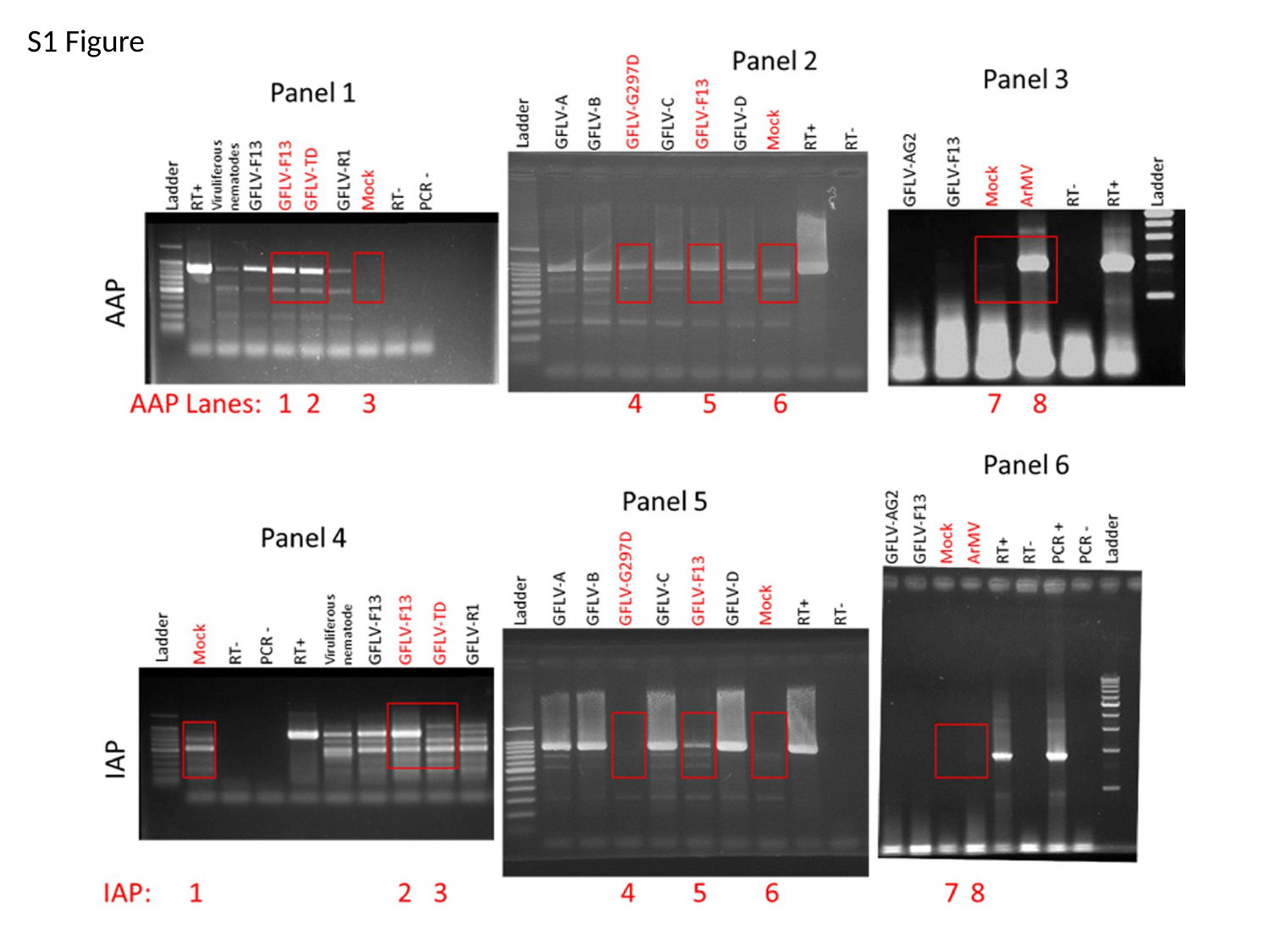

S1 Figure

Supplement: S1 Fig — The nematode transmission tests were performed in a greenhouse using aviruliferous X. index nematodes isolated from rearings established on fig plants (Ficus carica). Transmission test is a two-step procedure: an acquisition access period (AAP) and an inoculation access period (IAP). About 300 nematodes were given access to the roots of an infected Nicotiana benthamiana source plant. After an AAP of 6 weeks, infected source plant was replaced by a healthy N. benthamiana bait plant and grown in greenhouse for an IAP of 6 weeks. At the end of each step, nematodes were randomly collected. The presence of virus was verified in nematodes by reverse transcription-PCR (RT-PCR) with GFLV or ArMV-specific primers. Panels 1 to 3 below correspond to AAP analyses. Panel 4 to 6 below correspond to IAP analyses. Red boxes indicate the cropped area used to assemble the corrected Fig 1C with lanes indicated in red below the panels. (PPTX) [file ppat.1006268.s001.pptx]
